# CRISPR/CasRx suppresses KRAS-induced brain arteriovenous malformation developed in postnatal brain endothelial cells in mice

**DOI:** 10.1172/jci.insight.179729

**Published:** 2024-11-22

**Authors:** Shoji Saito, Yuka Nakamura, Satoshi Miyashita, Tokiharu Sato, Kana Hoshina, Masayasu Okada, Hitoshi Hasegawa, Makoto Oishi, Yukihiko Fujii, Jakob Körbelin, Yoshiaki Kubota, Kazuki Tainaka, Manabu Natsumeda, Masaki Ueno

**Affiliations:** 1 Department of Neurosurgery and; 2 Department of System Pathology for Neurological Disorders, Brain Research Institute, Niigata University, Niigata, Japan.; 3 Department of Oncology, Hematology and Bone Marrow Transplantation, University Medical Center Hamburg-Eppendorf, Hamburg, Germany.; 4 Department of Anatomy, Keio University School of Medicine, Tokyo, Japan.

**Keywords:** Neuroscience, Molecular pathology, Mouse models, Neurological disorders

## Abstract

Brain arteriovenous malformations (bAVMs) are anomalies forming vascular tangles connecting the arteries and veins, which cause hemorrhagic stroke in young adults. Current surgical approaches are highly invasive, and alternative therapeutic methods are warranted. Recent genetic studies identified *KRAS* mutations in endothelial cells of bAVMs; however, the underlying process leading to malformation in the postnatal stage remains unknown. Here we established a mouse model of bAVM developing during the early postnatal stage. Among 4 methods tested, mutant *KRAS* specifically introduced in brain endothelial cells by brain endothelial cell–directed adeno-associated virus (AAV) and endothelial cell–specific *Cdh5*-*CreERT2* mice successfully induced bAVMs in the postnatal period. Mutant *KRAS* led to the development of multiple vascular tangles and hemorrhage in the brain with increased MAPK/ERK signaling and growth in endothelial cells. Three-dimensional analyses in cleared tissue revealed dilated vascular networks connecting arteries and veins, similar to human bAVMs. Single-cell RNA-Seq revealed dysregulated gene expressions in endothelial cells and multiple cell types involved in the pathological process. Finally, we employed CRISPR/CasRx to knock down mutant *KRAS* expression, which efficiently suppressed bAVM development. The present model reveals pathological processes that lead to postnatal bAVMs and demonstrates the efficacy of therapeutic strategies with CRISPR/CasRx.

## Abstract 2

A novel mouse model of brain arteriovenous malformation reveals pathological processes during the postnatal stage and the efficacy of therapeutic strategies with CRISPR/CasRx.

## Graphical abstract

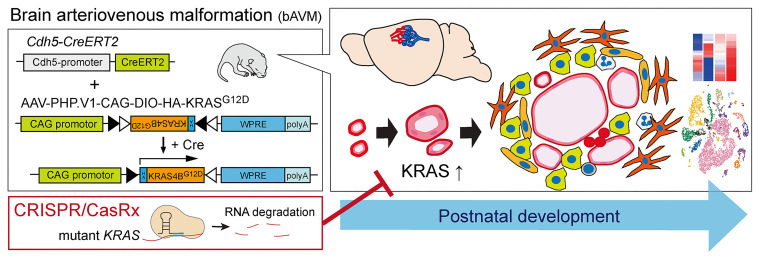

## Introduction

Brain arteriovenous malformations (bAVMs) are vascular anomalies that form vascular tangles directly connecting arteries and veins without capillary beds, called nidus (1, 2). The arteriovenous shunt elevates blood pressure and increases the risk of rupture and hemorrhage (3, 4). bAVMs affect approximately 10 per 100,000 individuals, causing 1%–2% of strokes and one-third of intracerebral hemorrhages in children and young adults. Current therapies include microsurgical resection, endovascular embolization, and stereotactic radiosurgery, leading to successful treatment in a majority of cases (1). However, the invasive surgery carries a high risk in cases in which the nidus is large or located in eloquent brain areas. A better understanding of the mechanisms underlying the malformation process might help to develop noninvasive therapeutic options.

Gene mutations such as *RASA1*, *ENG*, *ACVRL1*, and *SMAD4* have been identified in rare genetic syndromes causing vascular malformations (5). However, the causes of sporadic bAVMs without a family history, which comprise 95% of cases, remained largely unknown. Importantly, a recent study identified *KRAS* gene mutations in the endothelial cells of sporadic cases (6), showing 28%–87% of them harbor somatic mutation (6–12). *KRAS* encodes RAS GTPase, a downstream of tyrosine kinase receptors in RAF/MEK/ERK and PI3K/AKT/mTOR pathways (13, 14). The mutations at codon 12 (e.g., G12C, G12V, G12A, G12D) lead to constitutive activation by preventing GTP hydrolysis and induce aberrant cell growth and proliferation. Indeed, in vitro studies showed that *KRAS* mutants induce endothelial growth and endothelial-mesenchymal transition (EndMT) (6, 15). Recent studies have further shown that mutant *KRAS* induction in endothelial cells develops vascular tangles in mice and zebrafish, suggesting that the mutation is causative for bAVMs (16, 17).

bAVMs are assumed to develop congenitally or de novo postnatally (18, 19). Since bAVMs are noticed when the patients become symptomatic by hemorrhage or epilepsy in most cases (18, 20), the malformation process in the asymptomatic phase has not been fully understood. An appropriate animal model that develops in the postnatal stage will help investigate the process. The first animal study to develop KRAS mutant bAVM used brain endothelial cell–specific *Slco1c1*-*CreER* or endothelial cell–specific *Cdh5*-*CreER* and *lox*-*stop*-*lox*-*Kras^G12D^* (*lsl*-*Kras^G12D^*) mice to introduce mutant *Kras* in endothelial cells (16). They successfully formed aberrant vasculatures that resembled bAVMs; however, the phenotype and penetrance were weak (e.g., 50% at maximum), and Cre recombination was not completely restricted to the brain endothelial cells. A subsequent study induced mutant *Kras* in early postnatal days in *Cdh5*-*CreER*;*lsl*-*Kras^G12D^* mice; however, it affected survival, accompanied by vessel dilation in other organs due to systemic recombination (21). Another study used brain endothelial cell–directing adeno-associated virus (AAV) serotype BR1 to induce *KRAS^G12V^* at 5 weeks of age in mice (17). This method induced bAVM-like structures in the brain; however, it was technically difficult to induce mutant *KRAS* in the postnatal stage. Collectively, the pathological process in the postnatal stage remained unclear even in animal models. An alternative clinically relevant model developed in postnatal brain endothelial cells is required to study pathological mechanisms as well as to develop therapeutic methods.

Noninvasive therapeutic options such as pharmacological treatments have been tested in animal models. Since MAPK/ERK signals in endothelial cells, MEK/ERK inhibitors have been shown to suppress growth and transformation in culture (6). Subsequent studies have revealed that the inhibitors alleviated vascular tangle formation in zebrafish and mice (16, 17, 21). However, the treatments did not block the malformation completely. Additional approaches are required to suppress the pathological process.

In this study, we aimed to establish a mouse model that initiates bAVMs during the postnatal stage. We utilized brain endothelial cell–directing AAV combined with endothelial cell–specific *CreER* mice and developed a model that efficiently induced vascular tangles during the postnatal stage. This model highlights the malformation process and the efficacy of gene therapy with CRISPR/CasRx that suppressed mutant *KRAS* expression and malformation. The model will be useful for understanding the pathological process and developing therapeutic approaches for bAVMs.

## Results

### Development of a postnatal bAVM model by brain endothelial cell–specific induction of mutant KRAS.

**Figure 1:**
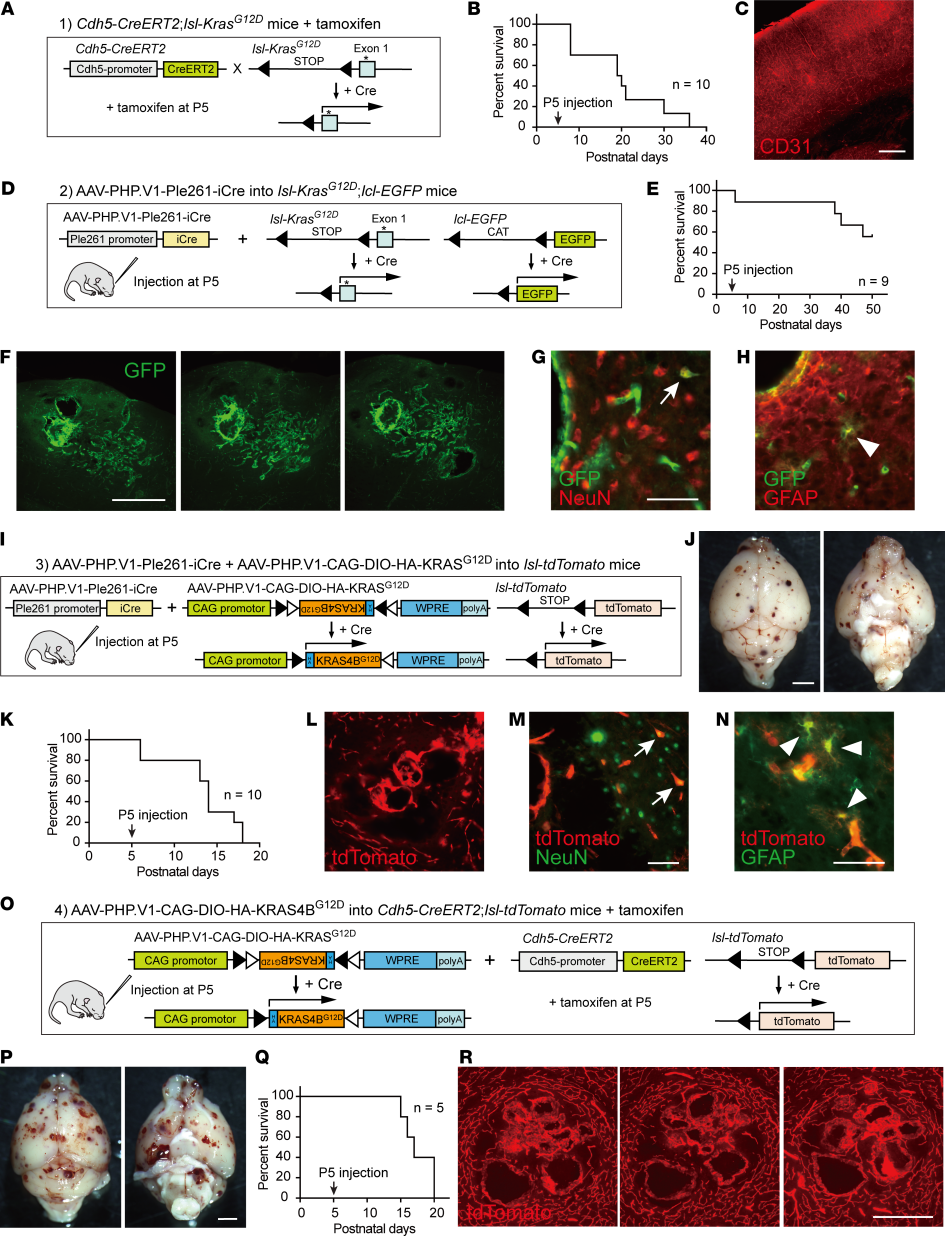
Development of mouse bAVM models in the postnatal stage. (**A**–**C**) A model of *Cdh5*-*CreERT2*;*lsl-Kras^G12D^* mice with tamoxifen injection at P5. (**B**) Survival curve (*n* = 10). (**C**) A representative image of the cerebral cortex with CD31 staining (red). (**D**–**H**) A model of AAV-PHP.V1-Ple261-iCre–injected *lsl*-*Kras^G12D^*;*lcl*-*EGFP* mice. (**E**) Survival curve (6 × 10^10^ GC, *n* = 9). (**F**) Representative serial brain section images of EGFP^+^ vascular tangle formation (green, P84). (**G** and **H**) Ectopic EGFP expressions in NeuN^+^ neurons (arrow) and GFAP^+^ astrocytes (arrowhead). (**I**–**N**) A model of AAV-PHP.V1-Ple261-iCre– and AAV-PHP.V1-CAG-DIO-HA-KRAS^G12D^–injected *lsl-tdTomato* mice. (**J**) The brain showing multiple hemorrhagic lesions (6 × 10^10^ GC, P15). (**K**) Survival curve (*n* = 10). (**L**) Brain section images of tdTomato^+^ vascular tangles (red, P15). (**M** and **N**) Ectopic tdTomato expressions in NeuN^+^ neurons (arrow) and GFAP^+^ astrocytes (arrowhead). (**O**–**R**) A model of AAV-PHP.V1-CAG-DIO-HA-KRAS^G12D^– and tamoxifen-injected *Cdh5*-*CreERT2*;*lsl*-*tdTomato* mice. (**P**) Brain images showing multiple hemorrhagic lesions (6 × 10^10^ GC, P18). (**Q**) Survival curve (*n* = 5). (**R**) Serial brain section images of tdTomato^+^ vascular tangles (red, P18). Scale bars: 250 μm (**C**), 500 μm (**F** and **R**), 50 μm (**G**, **H**, **M**, and **N**), 2 mm (**J** and **P**), 100 μm (**L**).

To develop a mouse bAVM model in the postnatal stage, we tested 4 different methods to restrict mutant *KRAS* expression to postnatal brain endothelial cells (Figure 1). We first generated mice harboring endothelial cell–specific *Cdh5*-*CreERT2* (22) and *lsl*-*Kras^G12D^* (*Cdh5*-*CreERT2*;*lsl*-*Kras^G12D^*), to introduce *Kras^G12D^* in endothelial cells in a Cre-dependent manner (Figure 1A). Tamoxifen injection at P5 resulted in death in most mice at around P20 (*n* = 10) (Figure 1B). Most of the brains did not show clear vascular changes such as dilation or hemorrhage, except for 1 mouse (9 of 10 mice; Figure 1C and Supplemental Figure 1, A and B; supplemental material available online with this article; https://doi.org/10.1172/jci.insight.179729DS1). Consistent with this, during our examination, another study reported a high mortality and low penetration of hemorrhage without vascular tangle formation in similar mouse lines (16). Thus, we did not pursue this model further.

We next tested AAVs to introduce exogenous genes in brain endothelial cells. We first examined the efficiency and specificity of 2 recently reported AAV variants that target brain endothelial cells: BR1 (23) and PHP.V1 (24). Either variant that expressed EGFP was injected retro-orbitally in neonates and was found to efficiently label brain endothelial cells (Supplemental Figure 1, C and D). However, they also frequently labeled astrocytes and neurons. This suggests that the previously reported minor leakiness of both variants in adults (23, 24) would increase when injected in early postnatal days. To further restrict the expressions, we selected one of the variants, PHP.V1, in the subsequent model development.

As the second model, we generated AAV-PHP.V1-Ple261-iCre that expressed Cre under endothelial cell–specific Ple261 promoter (24, 25), and we injected it into *lsl*-*Kras^G12D^*;*lo*x-*CAT*-*lox*-*EGFP* (*lcl*-*EGFP*) mice at P5, which would express *Kras^G12D^* and reporter EGFP in a Cre-dependent manner (Figure 1D). Although the mice showed gradual decrease of survival rate (Figure 1E), we found that aberrant EGFP^+^ vascular tangles with multiple dilated and plexiform vessels were formed in the brain at 6 weeks (Figure 1F and Supplemental Figure 1, E–G). This indicates that the method successfully induced a bAVM-like vasculature. Unexpectedly, however, EGFP expression was also occasionally observed in NeuN^+^ neurons and GFAP^+^ astrocytes (Figure 1, G and H), and this was also seen in control *lcl*-*EGFP* mice (Supplemental Figure 1, H–J). This suggests that this model induces *Kras^G12D^* in other cell types by a leaked Cre expression.

Third, we tested simultaneous injections of AAV-PHP.V1-Ple261-iCre and AAV-PHP.V1-CAG-DIO-HA-KRAS^G12D^ which expresses *KRAS^G12D^* by Cre recombination, into *lo*x-*stop*-*lox*-*tdTomato* (*lsl-tdTomato*) reporter mice (Figure 1I). Although the mice died by P18 (10 of 10 mice), it was found to induce multiple hemorrhagic spots in the brain (Figure 1, J and K). Moreover, a number of tdTomato^+^ dilated and plexiform vessels were found in brain sections (Figure 1L). However, leaky tdTomato^+^ expression was again found in neurons and astrocytes (Figure 1, M and N, and Supplemental Figure 1, K and L). This could be due to ectopic Cre expression as in the second model.

To restrict the *KRAS* expression more strictly, we lastly tested the injection of AAV-PHP.V1-CAG-DIO-HA-KRAS^G12D^ (6 × 10^10^ GC) into *Cdh5*-*CreERT2*;*lsl*-*tdTomato* mice with tamoxifen injection at P5 (Figure 1O). Although the mice died by P20 (*n* = 5), a number of hemorrhagic sites were induced in the brain (Figure 1, P and Q). Serial sections revealed that a number of abnormal tdTomato^+^ vascular tangles were formed (Figure 1R). Neurons or glial cells mostly did not express tdTomato, indicating high specificity of *KRAS^G12D^* induction in endothelial cells. Thus, the last model successfully and specifically induced bAVM-like lesions in the postnatal brain.

### Vascular changes in postnatally induced bAVM model.

**Figure 2:**
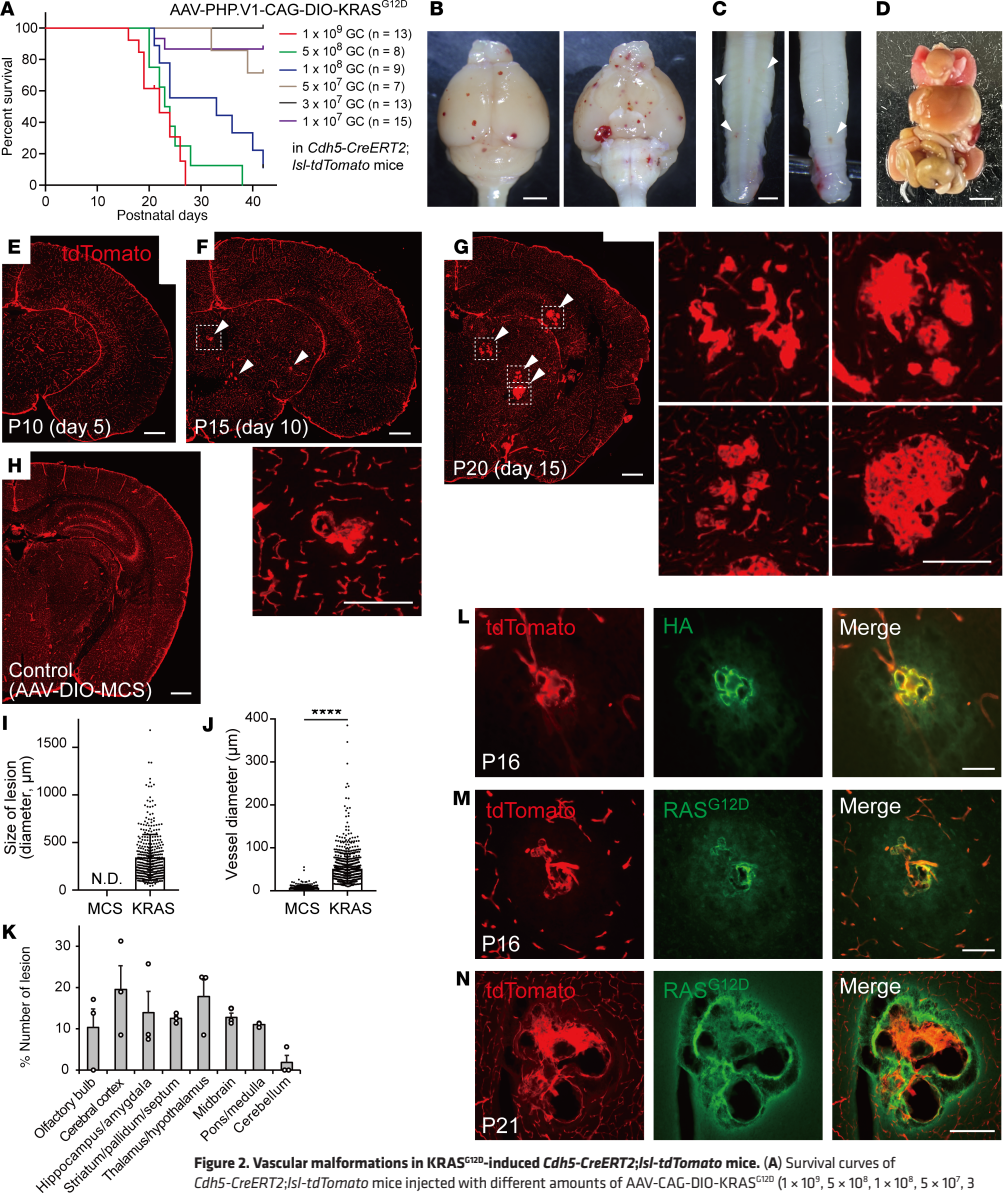
Vascular malformations in KRAS^G12D^-induced *Cdh5-CreERT2*;*lsl*-*tdTomato* mice. (**A**) Survival curves of *Cdh5-CreERT2*;*lsl*-*tdTomato* mice injected with different amounts of AAV-CAG-DIO-KRAS^G12D^ (1 × 10^9^, 5 × 10^8^, 1 × 10^8^, 5 × 10^7^, 3 × 10^7^, and 1 × 10^7^ GC). (**B**–**D**) Representative images of the brain (**B**), spinal cord (**C**), and organs (**D**) (1 × 10^9^ GC, P21). (**E**–**H**) Brain section images at days 5, 10, and 15 after AAV-PHP.V1-CAG-DIO-HA-KRAS^G12D^ (**E**–**G**) or control AAV-PHP.V1-CAG-DIO-MCS injection (**H**) in *Cdh5-CreERT2*;*lsl*-*tdTomato* mice (1 × 10^9^ GC; 3 to 4 images were combined in each panel). Arrowheads indicate vascular tangles. Dotted areas are magnified in the neighboring panels. (**I**) The size of lesions (1 × 10^9^ GC, total 380 vascular tangles, *n* = 3 brains, mean ± SD). N.D., not detected, in control AAV (MCS). (**J**) Inner vessel diameter in control AAV and of the lesions in KRAS-induced group. ****P < 0.0001. *n* = 357 and 862 vessels in 2 animals; data are shown as mean ± SD. Mann-Whitney *U* test was used. (**K**) The ratio of lesion numbers in brain regions (*n* = 3 brains). (**L**–**N**) HA and RAS^G12D^ expressions in the vascular tangles (P16 and P21). Scale bars: 2 mm (**B**), 1 mm (**C**), 5 mm (**D**), 500 μm (**E**–**H**), 100 μm (**L**–**N**, insets in **F**, **G**).

Due to the high specificity and efficiency, we pursued the last model to examine pathological changes. Since the low survival rate limits subsequent studies of pathology and therapeutic approaches, we reduced the amount of AAV from 1 × 10^9^ to 1 × 10^7^ GC. A lower amount increased the survival rate (Figure 2A), but it still efficiently induced hemorrhagic lesions, although the number decreased (Figure 2B; 1 × 10^9^ GC). There were no sex differences in lesion formation (Supplemental Figure 2, A and B). Hemorrhagic lesions were also observed in the spinal cord (Figure 2C), while they did not appear in other organs except for occasional hemorrhagic sites in the spleen (4 of 5 mice) (Figure 2D and Supplemental Figure 2D). Histological analyses revealed that a lower amount of AAV also generated a number of malformed vascular tangles in the brain (Figure 2G; 57.7 ± 13.0/brain, *n* = 3), while control AAV-PHP.V1-CAG-DIO-MCS injections did not (Figure 2H; 0 ± 0, *n* = 3; Supplemental Figure 2, A and C). The aberrant vascular structures gradually grew and became complex from day 5 to 15 after the injections (Figure 2, E–G). Most of the vascular tangles were 300–500 μm in size, and a few of them grew to over 1,000 μm (Figure 2I). The vessel diameters were enlarged in the tangles (Figure 2J). They were distributed throughout the brain, while the occurrence was somewhat lower in the cerebellum (Figure 2K).

We further examined whether the induced mutant *KRAS* actually generated vascular tangles. The expression of HA tagged with KRAS^G12D^ was mostly observed in tdTomato^+^ endothelial cells that formed vascular tangles (78.8% ± 7.72%, *n* = 3; Figure 2L). RAS^G12D^ was also strongly expressed in those tdTomato^+^ cells (Figure 2, M and N). HA^–^ endothelial cells were occasionally observed in the edge of the lesion, some of them with dilation (Supplemental Figure 2E), suggesting that the lesion was mainly formed by KRAS^G12D^-induced cells and connected with noninduced endothelial cells. Taken together, the data indicate that mutant *KRAS* is introduced specifically to endothelial cells and efficiently drives bAVM-like vasculature.

### Three-dimensional analyses revealed nidus formation in the bAVM model.

**Figure 3:**
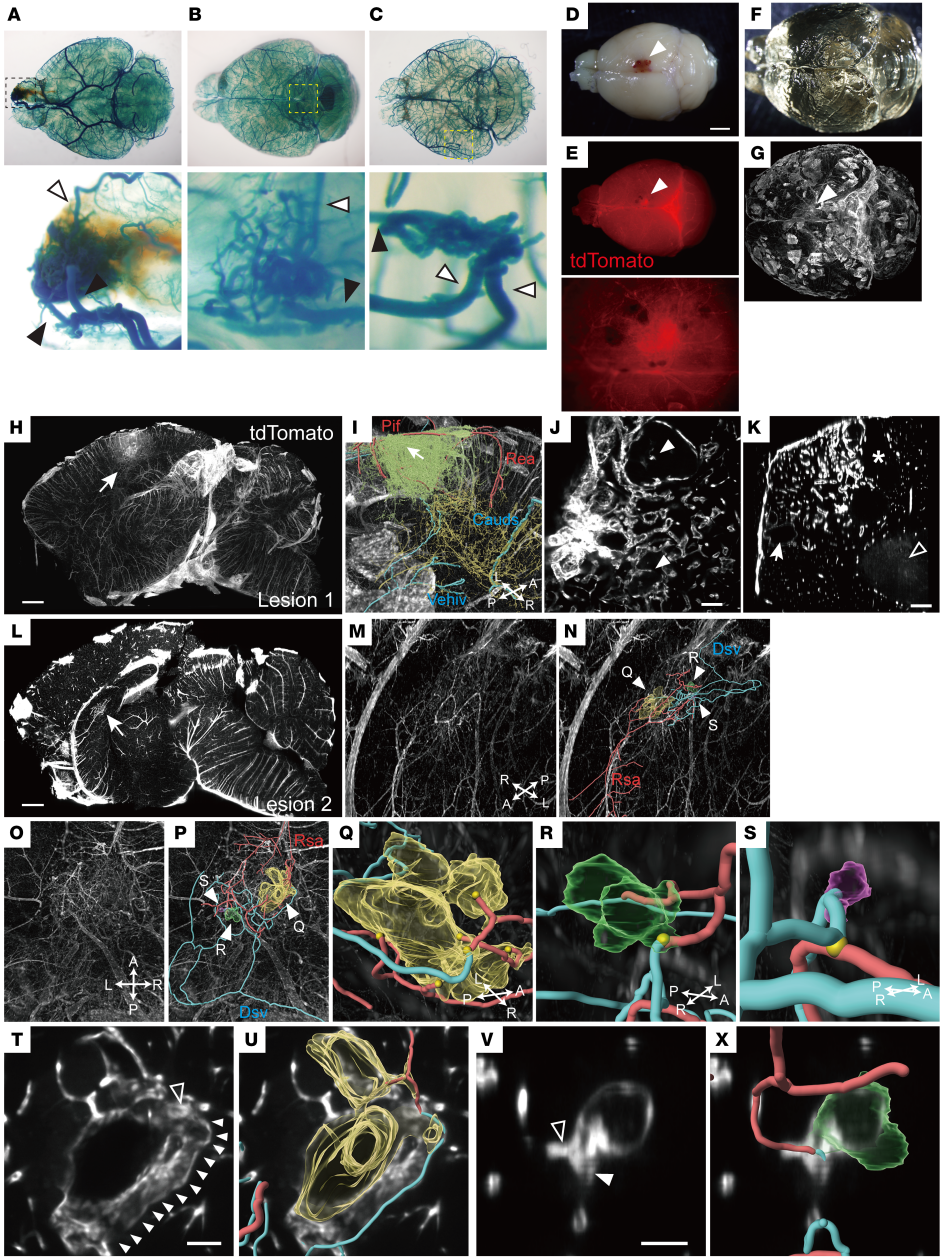
Three-dimensional analyses reveal a nidus structure in cleared brains of KRAS^G12D^-induced *Cdh5-CreERT2*;*lsl*-*tdTomato* mice. (**A**–**C**) Three representative cleared brains of latex-infused KRAS^G12D^-induced *Cdh5-CreERT2*;*lsl*-*tdTomato* mice (3 × 10^7^ GC, P42) with tangled vasculature. Bottom, magnified views of the dotted areas. Arrowheads, feeding arteries (white) and draining veins (black). (**D**–**G**) A representative brain image of KRAS^G12D^-induced *Cdh5-CreERT2*;*lsl*-*tdTomato* mice (**D**), fluorescence image in stereomicroscopy (**E**; bottom, magnified view of the lesion [arrowheads]), cleared brain by CUBIC (**F**), and light-sheet microscopic imaging (**G**). (**H** and **I**) Sagittal sectioning view by IMARIS (**H**) and 3D magnified view (**I**) of the lesion in the cerebral cortex (arrow). Green, vascular tangle; red, feeding arteries (Rea, retrosplenial artery; Pif, posterior internal frontal artery); blue, draining veins (Cauds, caudal branch of the superior sagittal sinus; Vehiv, ventral hippocampal vein). A, anterior; P, posterior; R, right; L, left. (**J** and **K**) Horizontal (**J**) and coronal (**K**) sectioning views of **I**. White arrowheads, vascular pillars in dilated vessels; arrow, dilated vein; asterisk, necrotic area; black arrowhead, hemorrhagic area. (**L**–**P**) Another sagittal view of **G** showing a vascular tangle in the septum (**L**, arrow) and 3D magnified sagittal (**M** and **N**) and horizontal images (**O** and **P**). Nidus structures (arrowheads, green/yellow/purple) connected with feeding arteries (red; Rsa, rostral septal artery) and draining veins (blue; Dsv, dorsal septal vein). (**Q**–**S**) Magnified views of arteriovenous junctions (arrowheads in **N** and **P**), showing dilated vessels connected with arteries and veins (**Q**) and aneurysms (**R** and **S**). Yellow circles, arteriovenous junctions. (**T–X**) Sectioned views of dilated vessels in **Q** and aneurysm in **R** (**T** and **V**) with 3D images of dilated vessels (yellow, green), arteries (red), and veins (blue) (**U** and **X**). Arrowheads, vein; black arrowheads, artery. Scale bars: 2 mm (**D**), 1 mm (**H**, **L**), 200 μm (**J**, **K**, and **T**–**X**).

A nidus is defined as a vascular structure shunted with the feeding arteries and draining veins (4, 26). We next examined if the vascular tangles in the present model contained structural features of the nidus. We analyzed the model with 3 × 10^7^ GC virus injections, in which mice survived until adulthood with only a few lesions in the brain (1.88 ± 0.40/brain, *n* = 8; Figure 2A, Figure 3D, and Supplemental Figure 3A). Under a fluorescence stereomicroscope, we observed that the hemorrhagic area had tdTomato^+^ vascular tangles that were supplied by multiple feeding arteries and drained into dilated veins (Supplemental Figure 3B). We further visualized the vasculature by blue latex infusion (17, 27). Tortuous and enlarged vascular tangles connected with feeding arteries and draining veins were observed in the cleared brains (Figure 3, A–C), indicating a direct flow between the arteries and veins.

To determine the structure in detail, we further examined 3D structures in cleared brains by a tissue-clearing technique, CUBIC (28) (Figure 3, D–F). Light-sheet microscopic imaging revealed tdTomato^+^ vascular tangles comprising tortuous and plexiform vessels (Figure 3, G, H, and L). We especially analyzed 2 representative tangles that were found in the brain of Figure 3D.

Lesion 1 was located at the surface of the cerebral cortex (Figure 3, H–K, and Supplemental Video 1). Referring to an anatomical vascular atlas of the mouse brain (29), we identified that the vascular tangle was fed by the retrosplenial artery (Rea) and posterior internal frontal artery (Pif), and was drained into the caudal branch of the superior sagittal sinus (Cauds) (Figure 3I). In this lesion, multiple vessels were layered around the large dilated vessel, as seen in human bAVMs (Figure 3, J and K, and Supplemental Figure 3C; ref. 30). Intravascular pillars were occasionally observed within the dilated vessels (Figure 3J), which implicated abortive intussusceptive angiogenesis as reported in zebrafish cerebral cavernous malformations (31). Some parts of the lesion lost tdTomato^+^ signals, which would correspond to hemorrhagic or necrotic areas (Figure 3K), and made it difficult to fully trace the vascular connections.

Lesion 2 was found in the septum (Figure 3L, Supplemental Figure 3D, and Supplemental Video 2). The tangle was fed by the rostral septal artery (Rsa) and drained by the thalamostriate to the dorsal septal vein (Dsv; Figure 3, M–P; ref. 29). Three-dimensional analyses revealed that the lesion contained 3 parts of direct arteriovenous connections (Figure 3, N and P). The largest part had 4 feeding arteries around the arteriovenous junction, and 2 more arteries were fed into the enlarged part of the vein (Figure 3Q). Multiple locations were recurrently joined to each other in the veins, while the fusions were not observed in the arteries. The second part had a saccular aneurysm-like vessel at the arteriovenous junction (Figure 3R), as frequently observed in human bAVMs (4). The last part also formed a varicose-like structure close to the vein at the arteriovenous junction (Figure 3S). In all the cases, the draining veins were dilated compared with the arteries. Image sectioning of the dilated vessels showed enlarged vasculatures, similar to the histological images (Figure 1R and Figure 2N). Direct arteriovenous connections (Figure 3, T–X) and intravascular pillars were also observed in the sectioned images.

Taken together, we confirmed that the vascular tangle had direct connections with multiple afferent arteries and dilated draining veins, indicating that a nidus was formed in this model. The 3 dilated arteriovenous junctions of different sizes in lesion 2 likely represent the malformation process that finally develops into a large-sized nidus as in lesion 1, causing ruptures and hemorrhage.

### Histopathological changes in the process of vascular malformation.

**Figure 4:**
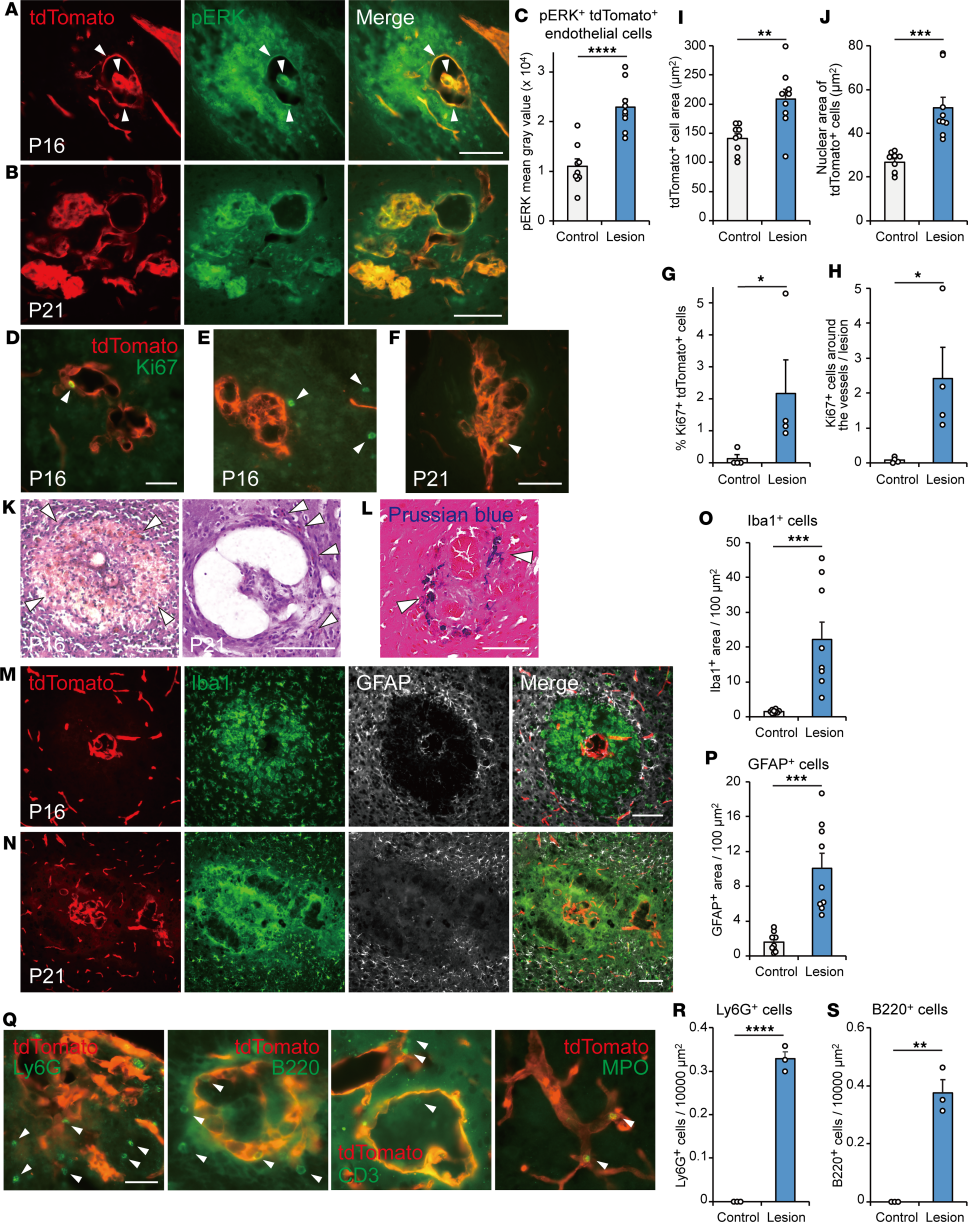
Histopathological changes in the lesions of KRAS^G12D^-induced *Cdh5-CreERT2*;*lsl*-*tdTomato* mice. (**A** and **B**) Representative images of pERK expression in tdTomato^+^ endothelial cells in the lesion of AAV-DIO-KRAS^G12D^ and tamoxifen-injected *Cdh5-CreERT2*;*lsl*-*tdTomato* mice (1 × 10^9^ GC, P16, P21). (**C**) Quantified pERK expression in tdTomato^+^ endothelial cells in the lesion and adjacent control area. *n* = 9 lesions in 3 animals, unpaired *t* test. (**D**–**F**) Ki67 expression in the lesion (arrowheads, P16, P21). (**G** and **H**) Quantification of Ki67^+^ cells in tdTomato^+^ endothelial cells (%) and Ki67^+^ tdTomato^–^ cells in the lesion and adjacent control area. *n* = 4 animals, Mann-Whitney *U* test (**G**) and unpaired *t* test (**H**). (**I** and **J**) Cell and nuclear area of tdTomato^+^ endothelial cells in the lesion and adjacent control area. *n* = 9 lesions in 3 animals, unpaired *t* test (**I**) and Mann-Whitney *U* test (**J**). (**K**) H&E staining showing hemorrhage and hemosiderin-laden macrophages/microglia (arrowheads) in the lesion (P16, P21). (**L**) Iron staining with Prussian blue in the lesion (blue, arrowheads). (**M** and **N**) Activated Iba1^+^ microglia/macrophages and GFAP^+^ astrocytes surrounding the lesion (P16, 21). (**O** and **P**) Quantification of Iba1^+^ (**O**) and GFAP^+^ cell areas (**P**) in the lesion and adjacent control area. *n* = 9 lesions in 3 animals, unpaired *t* test. (**Q**) Ly6G^+^ neutrophils, B220^+^ B cells, CD3^+^ T cells, and MPO^+^ neutrophils in the lesion (arrowheads). (**R** and **S**) Quantification of Ly6G^+^ (**R**) and B220^+^ cells (**S**) in the lesion and adjacent control area. *n* = 3 animals, unpaired *t* test. *P < 0.05, **P < 0.01, ***P < 0.001, ****P < 0.0001. Scale bars: 100 μm (**A**, **B**, **D**–**F**, and **K**–**N**), 20 μm (**Q**).

Previous studies have shown that mutant KRAS activates the MAPK/ERK pathway in endothelial cells (6, 16, 17). Consistently, pERK expression was increased in endothelial cells of the tangles in our model (Figure 4, A–C). A proliferation marker, Ki67, was occasionally seen in the tdTomato^+^ endothelial cells of the tangles and the cells surrounding it (Figure 4, D–H). The size of endothelial cells and nuclei were found to be larger in the lesion (Figure 4, I and J). These suggest that mutant KRAS activates the MAPK pathway and promotes the growth and proliferation of endothelial cells. Intravascular pillars were occasionally observed (Figure 4, A and Q) as in the 3D imaging (Figure 3J).

The vascular tangle occasionally formed cell-scarce or necrotic areas around it, which would correspond to microhemorrhagic sites. Indeed, H&E staining revealed RBCs and hemosiderin-laden macrophages/microglia in the parenchyma surrounding the enlarged vessels (Figure 4K), which were colored with iron staining (Figure 4L). Iba1^+^ microglia or macrophage accumulated there that were surrounded by reactive GFAP^+^ astrocytes (Figure 4, M–P). Ly6G^+^ neutrophils having segmented nuclei and B220^+^ B cells were also observed around the enlarged vessels (Figure 4, Q–S). CD3^+^ T cells and MPO^+^ neutrophils were also occasionally seen in or around the vessels (Figure 4Q), indicating inflammatory responses in the lesion. Collectively, the present model revealed various pathological events in the process of vascular malformation.

### Gene expression changes in multiple cell types in bAVM.

**Figure 5:**
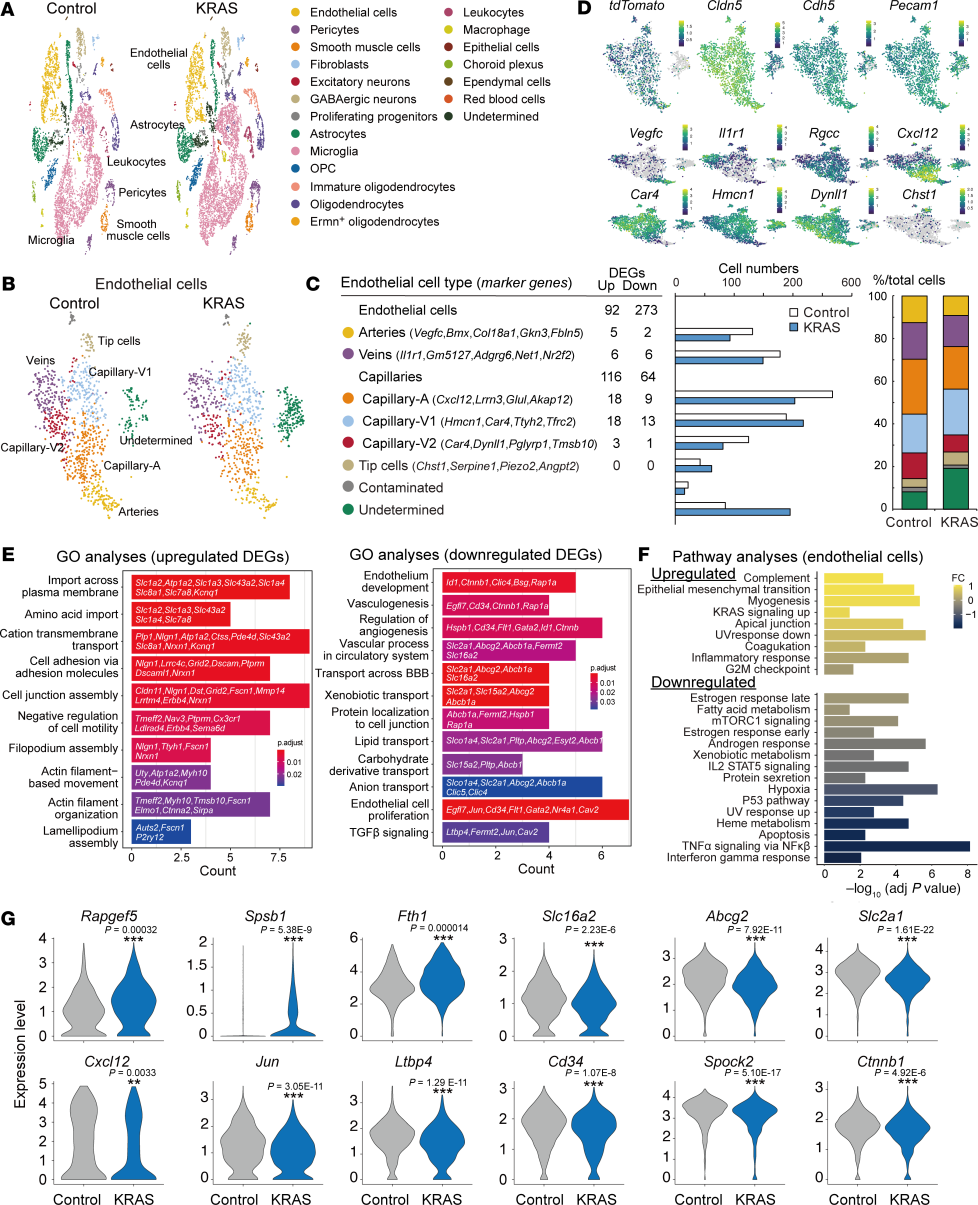
Single-cell RNA-Seq analyses of brain endothelial cells in KRAS^G12D^-induced mice. (**A**) t-SNE plot of the total extracted brain cells of AAV-CAG-DIO-MCS–injected (control) and AAV-CAG-DIO-KRAS^G12D^–injected(KRAS-injected) *Cdh5-CreERT2*;*lsl*-*tdTomato* mice. (**B**) t-SNE plot of endothelial cells in control and KRAS-group. (**C**) Representative gene markers, the number of up- and downregulated DEGs, cell numbers (middle), and ratio (right) of each endothelial cell type in control and KRAS-group. (**D**) tdTomato and endothelial cell type marker expressions in t-SNE plot. (**E**) Representative GO terms in up- and downregulated DEGs (KRAS versus control). (**F**) Pathway analyses of genes expressed in endothelial cells (KRAS versus control). (**G**) Violin plots of representative DEGs expressions in endothelial cells of control and KRAS groups, which were commonly observed in human bAVMs (32). **P < 0.01, ***P < 0.001.

We next preformed single-cell RNA-Seq (scRNA-Seq) to examine changes in cell types and gene expressions in bAVM development. tdTomato^+^ cells in the cerebral cortex were sorted to preferentially analyze endothelial cells and were further combined with tdTomato^–^ cells for other cell types. A total of 16,747 cells (control, 7,382 cells; KRAS group, 9,365 cells) were extracted and classified into cell types in t-distributed stochastic neighbor embedding (t-SNE) plots (Figure 5A and Supplemental Figure 4, A and B). *Cldn5^+^, Cdh5^+^,* and *Pecam1^+^* endothelial cells were first classified into 8 clusters, comprising *Vegfc*^+^ arteries; *Il1r1*^+^ veins; *Rgcc*^+^ capillaries, which include capillary-arterial (capillary-A) capillary-venous 1 (capillary-V1), and capillary-venous 2 (capillary-V2); and *Chst1*^+^ tip cells (Figure 5, B–D, Supplemental Figure 4C, and Supplemental Table 1; refs. 32–36). One cell cluster that had lower numbers of mRNAs and increased mitochondria-related genes was defined as “undetermined” cells (Supplemental Figure 4D), which might correspond to low quality or dying cells (Figure 5, B and C). Another cluster that expressed pericyte markers (e.g., *Anpep2*, *Kcnj8*) with increased numbers of detected mRNAs was defined as “contaminated” cells (Figure 5, B and C, Supplemental Figure 4D, and Supplemental Table 1; ref. 33). Capillary cell numbers showed a tendency to decrease in the *KRAS*-induced group, while those of tip and “undetermined” cells increased (Figure 5C). This is consistent with the decrease of capillaries in human bAVMs (32) and the increase of tip cells in HRAS-activated endothelial cells (37), though the present study was inapplicable for statistical comparison due to the small sample trials.

We then examined differentially expressed genes (DEGs) in the KRAS group compared with controls in each endothelial cell type. The capillaries exhibited a substantial number of up- and downregulated DEGs (116 and 64 genes, respectively), whereas minimal changes occurred in arteries and veins (Figure 5C and Supplemental Table 2). This suggests that the capillaries were mainly affected by *KRAS^G12D^* induction. GO analyses showed upregulated genes involved in transmembrane transport, cell adhesion, and motility in capillaries, whereas genes related to endothelial cell development, proliferation, and differentiation were paradoxically downregulated, as well as those in blood-brain barrier (BBB) transport and TGF-β signaling (Figure 5E and Supplemental Table 3). Pathway analyses further revealed upregulated genes in KRAS signaling (Figure 5F). Upregulated DEGs included genes in cytoskeletal and actin filament process (*Tmeff2*, *Tmsb10*, *Fscn1*), extracellular matrix and adhesion (*Pcdh9*, *Spsb1*, *Tnr*), cell growth (*Rapgef5*), and transporters (*Slc1a2*, *Atp1a2*, *Slc1a4*, *Slc43a2*, *Slc7a8*, *Slc1a3*, *Slc8a1*) (Supplemental Table 2). In contrast, many of the genes known as markers of brain endothelial cells related to transmembrane proteins, transcription factors, extracellular matrix, and transport across BBB (*Sema3c*, *Emcn*, *Slco1a4*, *Prom1*, *Id3*, *Tuba1b*, *Id1*, *Slc2a1*, *Cd34*, *Itm2a*, *Epas1*, *Spock2*, *Bsg*) (34, 38) were downregulated. Markers of brain endothelial cells (*Slc6a6*, *Pllp*, *Degs2*) and capillaries (*Itm2a*, *Cxcl12*) (34) were also decreased when compared in total endothelial cells. This suggests that homeostatic functions of endothelial cells might be disrupted. We further compared the scRNA-Seq data with those of human bAVM (32) and found 22 DEGs commonly upregulated (e.g., *Rapgef5*, *Spsb1*, *Tmsb10*, *Rftn1*, *Fth1*) and 87 downregulated (e.g., *Cxcl12*, *Jun*, *Ltbp4*, *Slc16a2*, *Abcg2*, *Slc2a1*, *Cd34*, *Spock2*, *Ctnnb1*) (Figure 5G and Supplemental Table 4). We could not detect changes in up- (*Plvap*, *Pgf*, *Ccl14*, *Stc1*, *Angpt2*) and downregulated (*Mfsd2a*, *Slc16a1*, *Slc38a5*) endothelial markers in human nidus cluster (32) (Supplemental Figure 4M). This suggests that the present model partially recapitulates the pathological process of human bAVMs, in which commonly disrupted genes might be essential for the pathogenesis. We validated the expressions of RAPGEF5 and SPSB1 by IHC. RAPGEF5 was strongly expressed in the nucleus of tdTomato^+^ endothelial cells in the lesion, while the expression was weak in those of adjacent control areas (Supplemental Figure 4, E–H). SPSB1 expression was also increased in a part of the vessels in the lesion, while it was mostly absent in adjacent endothelial cells (Supplemental Figure 4, I–L).

**Figure 6:**
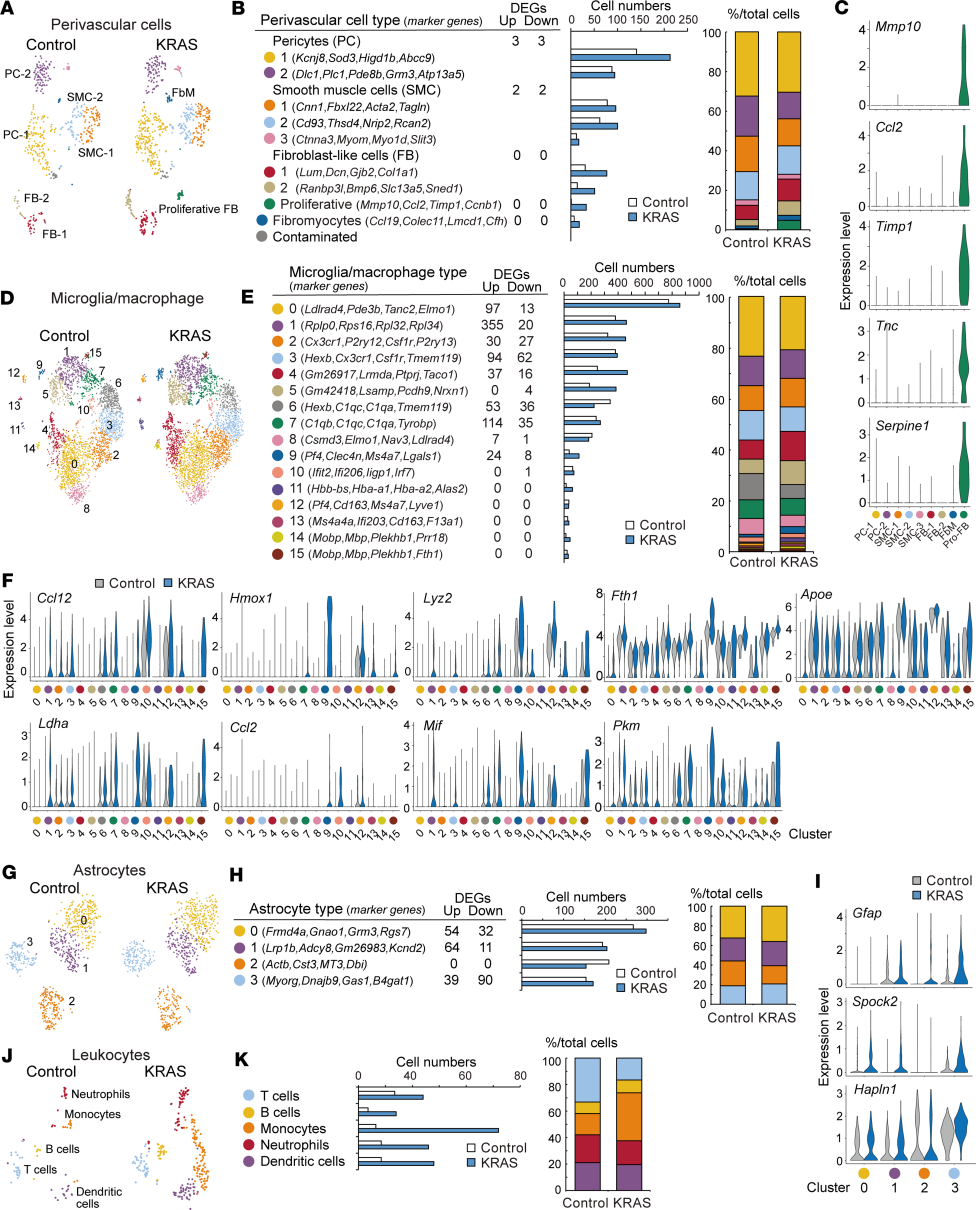
Single-cell RNA-Seq analyses of perivascular, glial, and immune cells in KRAS^G12D^-induced mice. (**A**) t-SNE plot of perivascular cells of AAV-CAG-DIO-MCS– (control) and AAV-CAG-DIO-KRAS^G12D^–treated (KRAS-treated) *Cdh5-CreERT2*;*lsl*-*tdTomato* mice. (**B**) Representative gene markers, the number of up- and downregulated DEGs, cell numbers (middle), and ratio (right) of each perivascular cell type in control and KRAS-group. (**C**) Violin plots of marker gene expressions of proliferative fibroblast-like cells (FB). (**D**) t-SNE plot of microglia/macrophage in control and KRAS group. (**E**) Representative gene markers, the number of up- and downregulated DEGs, cell numbers (middle), and ratio (right) of each microglia/macrophage cluster in control and KRAS group. (**F**) Violin plots of representative DEGs in microglia/macrophage clusters in control and KRAS group. (**G**) t-SNE plot of astrocytes in control and KRAS group. (**H**) Representative gene markers, the number of up- and downregulated DEGs, cell numbers (middle), and ratio (right) of each cluster of astrocytes in control and KRAS group. (**I**) Violin plots of representative DEGs in astrocyte clusters in control and KRAS group. (**J**) t-SNE plot of leukocytes in control and KRAS group. (**K**) Cell numbers (left) and ratio (right) of leukocytes in control and KRAS group.

Perivascular cells might also be involved in the pathogenesis (39). They were classified into *Pdgfrb^+^* pericytes, *Tagln*^+^ and *Acta2^+^* smooth muscle cells, *Dcn^+^* fibroblast-like cells, and *Ccl19*^+^ cells, which might correspond to fibromyocytes reported in human brains (32) (Figure 6, A and B; Supplemental Figure 5, A and B; and Supplemental Table 5). Intriguingly, we determined a new cell cluster that appeared in the KRAS group (Figure 6, A and B). The cells expressed transcripts related to cellular response to environmental stimulus and extracellular matrix (*Mmp10*, *Mmp9*, *Timp1*, *Tnc*) and mitosis (*Ccnb1*, *Spc24*, *Racgap1*, *Mki67*) as well as fibroblast-like cell markers (*Col6a3*, *Col5a3*, *Emp1*, *S100a6*) (33) (Figure 6C, Supplemental Figure 5C, and Supplemental Tables 5 and 6). Although they also mildly expressed pericyte and smooth muscle cell markers such as *Pdgfrb* and *Tagln* (Supplemental Figure 5A), we propose that they correspond to proliferative fibroblast-like cells.

We further examined microglia (*Cx3cr1*^+^, *P2ry12*^+^)/macrophage (*Msr1*^+^, *Mrc1*^+^) and astrocytes (*Aldh1l1*^+^), which were activated around the vascular tangles (Figure 4, M and N). Microglia/macrophages were found to comprise 16 clusters (Figure 6, D and E, and Supplemental Table 7). Clusters 2, 3, and 6 expressed homeostatic microglia markers (*Cx3cr1*, *P2ry12*, *Csf1r*, *Tmem119*) in addition to *Hexb*, *Ifngr1*, *C1qa*, and *C1qc*. Other clusters had specific gene profiles such as ribosomal proteins in 0, complement in 7, and IFN-induced genes in 10. Clusters 11 and 14/15 were enriched with erythroid (*Hbb*, *Hba*, *Alas2*) and oligodendrocytes-related genes (*Mobp*, *Mbp*, *Plekhb1*), respectively, which would correspond to phagocytosing cells (40) such as those containing hemosiderin around the lesion (Figure 4, K and L). Cluster 9, 12, and 13 expressed *Mrc1*, a marker of brain border or CNS-associated macrophages (BAMs or CAMs), in which dural (*Lgals3*, *Crip1*, *Vim*; cluster 9) and subdural BAM markers (*Lyve1*, *Colec12*, *Ednrb*; cluster 12 and 13) were preferentially expressed (41, 42). Cell numbers of clusters 4, 5, 9, 11, and 14 were increased, while cluster 6 was decreased, which may correspond to increased P2RY12^−^ cells in human bAVM (32) (Figure 6E). Many genes were upregulated in specific clusters (Figure 6E and Supplemental Table 8). The genes such as in translation, metabolic process, proteolysis, cytokines, and immune responses were broadly upregulated, while those in small GTPase, migration, TGF-β signaling, vascular control, and wound healing were decreased (Supplemental Figure 5D). For example, the transcripts in chemokines, phagocytosis, and metabolic processes (*Ccl12*, *Hmox1*, *Ifi30*, *Lyz2*, *Fth1*, *ApoE*, *Tspo*, *Ldha*, *Ccl2*, *Mif*, and *Pkm*) were upregulated (Figure 6F), while homeostatic microglial genes *Ppfia4*, *Gpr34*, *Tgfbr1*, *P2ry12*, and *Cx3cr1* were downregulated (Supplemental Table 8).

Astrocytes were separated into 4 clusters (43, 44) (Figure 6, G and H, and Supplemental Table 9). The cell numbers were not remarkably changed (Figure 6H). The clusters 0, 1, and 3 had substantial numbers of DEGs (Figure 6H and Supplemental Table 10). The genes involved in translation were upregulated, while those in cell matrix adhesion and vascular and BBB maintenance were decreased (Supplemental Figure 5E). In cluster 3, transcripts generally observed in reactive astrocytes such as *Gfap* (cytoskeleton), *Spock2* and *Hapln1* (extracellular matrix), and *C4b* (complement) were increased (Figure 6I), which would correspond to GFAP^+^ cells increased around the lesion (Figure 4, M, N, and P).

Lastly, an increase in leukocyte numbers was found in the KRAS group, including T cells (*Cd3e*^+^, *Cd3d*^+^), B cells (*Ms4a1*^+^, *Cd19*^+^), monocytes (*Ms4a4a*^+^, *Msr1*^+^), neutrophils (*Cxcr2*^+^, *Nlrp12*^+^), and DCs (*Cd209a*^+^) (Figure 6, J and K, and Supplemental Figure 4B). This is consistent with the histological observation (Figure 4, Q–S). Taken together, the scRNA-Seq data reveal aberrant changes in gene expression and multiple–cell type activation beyond the endothelial cells, which would all be involved in the pathogenesis.

### Knockdown of mutant KRAS by CRISPR/CasRx suppresses bAVMs.

**Figure 7:**
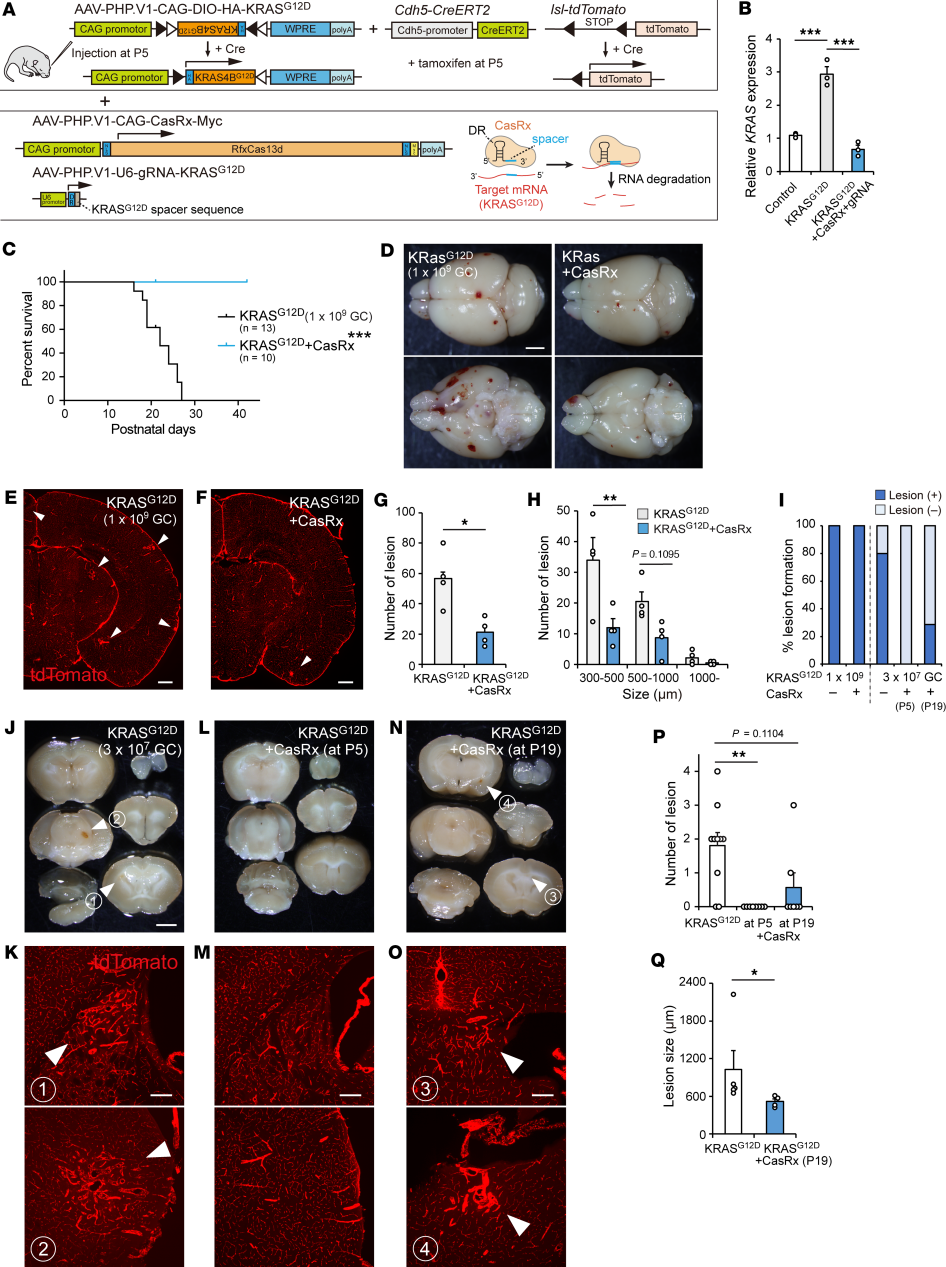
*KRAS* knockdown by AAV delivery of CRISPR/CasRx suppressed bAVM development. (**A**) A design of CRISPR/CasRx to silence *KRAS^G12D^* mRNA expression. DR, direct repeat. The right bottom image was modified from refs. 45, 47. (**B**) *KRAS* mRNA expressions in HEK293T cells transfected with control, *KRAS^G12D^*, and *KRAS^G12D^*-targeting CRISPR/CasRx plasmids in real-time PCR. *n* = 3, 1-way ANOVA followed by Tukey’s test. (**C**) Survival curves of KRAS^G12D^-induced (1 × 10^9^ GC) and *KRAS^G12D^*-CasRx–treated (1 × 10^10^ GC) mice. *n* = 13 and 10, log-rank test. (**D**) Brain images of *KRAS^G12D^*-induced (1 × 10^9^ GC) and CasRx-treated mice at P21. (**E** and **F**) Representative images of vascular tangle formation (arrowheads) in KRAS^G12D^-induced (1 × 10^9^ GC, **E**) and CasRx-treated (**F**) mice. (**G** and **H**) Lesion numbers (**G**) and size (**H**) in KRAS^G12D^-induced (1 × 10^9^ GC) and CasRx-treated mice. *n* = 4, unpaired *t* test (**G**), 2-way repeated ANOVA followed by Bonferroni’s test (**H**). (**I**) Percentage of mice forming lesions in KRAS^G12D^-induced (1 × 10^9^ GC, *n* = 13; 3 × 10^7^ GC, *n* = 10) and CasRx-treated mice (*n* = 10, 8, 7). (**J**–**O**) P42 brains of *KRAS^G12D^*-induced (3 × 10^7^ GC, **J** and **K**) and CasRx-treated mice (**L** and **M**, treated at P5; **N** and **O**, treated at P19). Bottom panels (**K**, **M**, and **O**) show histological images of vascular tangles in the upper panels (arrowheads, **J** and **N**) or corresponding areas (**M**). (**P** and **Q**) Lesion numbers (**P**) and size (**Q**) in KRAS^G12D^-induced (3 × 10^7^ GC) and CasRx-treated mice. *n* = 10, 8, 7, Kruskal-Wallis test followed by Dunn’s multiple-comparison test (**P**); *n* = 5, 4, Mann Whitney *U* test (**Q**). ***P < 0.001, **P < 0.01, *P < 0.05. Scale bars: 2 mm (**D**, **J**, **L**, and **N**), 500 μm (**E** and **F**), 200 μm (**K**, **M**, and **O**).

Finally, we tested a therapeutic method to suppress bAVM development. As MEK inhibitors partially inhibited vascular malformation (6, 16, 17), we searched for an alternative method to target upstream molecules or directly inhibit mutant KRAS. We focused on a CRISPR/CasRx system that degrades specific target sequences of RNA more specifically than RNA interference (45, 46), which would enable us to target mutated sequence of *KRAS* with minimal off-target effects (Figure 7A). The knockdown effect of gRNA covering the mutated nucleoside A sequence of *KRAS^G12D^* (47) was first evaluated in cultured cells by expressing KRAS^G12D^, CasRx (RfxCas13d), and gRNA-*KRAS^G12D^* with plasmids. PCR analysis showed that CasRx and the gRNA significantly reduced *KRAS* expression to the control levels (Figure 7B). This suggests that CRISPR/CasRx degrade overexpressed mutant *KRAS* but minimally degrade the endogenous *KRAS*, although the primers could not discriminate both *KRAS* transcripts. We then made AAV vectors expressing CasRx and the gRNA-*KRAS^G12D^* and injected them into KRAS^G12D^-induced *Cdh5*-*CreERT2*;*lsl*-*tdTomato* mice. Treatment with 1 × 10^10^ GC of AAV-CasRx and gRNA vectors simultaneously with *KRAS^G12D^* induction (1 × 10^9^ GC) significantly increased the survival rate of mice (Figure 7C). Although all the CasRx-induced mice still had hemorrhagic lesions and vascular tangles, the number and size significantly decreased (Figure 7, D–I). We further tested the efficacy in a clinically relevant model that developed a few niduses with the lower 3 × 10^7^ GC of AAV (Figure 3 and Figure 7, J and K). In this case, simultaneous treatment with the AAV-CasRx and gRNA vectors at P5 completely blocked lesion formation (Figure 7, I, L, M, and P). We compared the therapeutic effects with the MEK inhibitor trametinib (17, 21). Two weeks of trametinib treatment showed a tendency to decrease lesion number, but it was not significant, while it suppressed body growth and hair development with wooly hair (Supplemental Figure 6), as reported in a human case (48). This indicated superior therapeutic effects of CasRx. Finally, AAV-CasRx and gRNA vectors were treated 14 days after KRAS^G12D^ induction when the lesion had begun to develop. The lesion number showed a tendency to decrease, and the size significantly decreased (Figure 7, I and N–Q). These results indicate that the viral gene delivery approach with CRISPR/CasRx is effective to suppress bAVM development.

## Discussion

The present study established a bAVM model that developed vascular tangles with nidus structure during the early postnatal period. This enabled us to examine the postnatal process of malformation, showing a multitude of changes in gene expressions and activations in endothelial and surrounding cells. It further revealed a therapeutic effect of mutant *KRAS* knockdown with gene delivery of CRISPR/CasRx to suppress malformations. Thus, the model will provide a useful platform to elucidate pathological mechanisms and develop therapeutic approaches for bAVMs.

### Postnatal bAVM model compared with other models.

bAVMs are believed to develop during pregnancy or de novo postnatally (18–20, 49–52). The present model is therefore clinically relevant, as the malformation is initiated during the postnatal stage, and enables the analysis of pathological processes even in the asymptomatic phase. Previous reports of *Cdh5*-*CreER* or *Slco1c1*-*CreER*;*lsl*-*Kras^G12D^* mice showed weak phenotype and penetrance to form bAVM-like vasculatures (16). *Cdh5*-*CreER*;*lsl*-*Kras^G12D^* mice showed minimal survival with vascular dilations in multiple organs (21). KRAS^G12V^ or MAP3K3 introduction by AAV-BR1 led to vascular malformation (17, 53), but they had difficulty in gene introduction, specificity, and lethality to generate bAVMs neonatally. We overcome these limitations by combining the usage of brain endothelial cell–directed AAV with endothelial cell specific Cre mice.

Optimizing the amount of virus further made the model flexible to control disease severity regarding the lesion number and survival rate. For example, a higher amount of virus generated abundant vascular tangles in 2 weeks and enabled the analysis of pathological process efficiently. A lower amount induced a focal bAVM with low mortality, which is more similar to those in human bAVM in which a single lesion is generally formed with a long asymptomatic phase (49). It should be noted that the lesion number in the high-amount virus group was remarkably higher compared with the models induced in young adults (5 at most in refs. 16, 17), suggesting that neonatal brains or endothelial cells are more susceptible to develop vascular malformation in response to KRAS.

Since the present model induced lesions in random locations, possibly due to stochastic places of virus infection, methodology to induce malformation in any preferred area will further help the analyses. For example, a cortical lesion will enable to use 2-photon microscopy to sequentially observe the malformation process and therapeutic effects in the same animal. We tested several methods to control the lesion position, such as a direct AAV injection into a target area or magnetic attraction of the circulating virus; however, they all failed to induce malformation in the intended area. Alternative methods could be explored in the future.

### Structural features of bAVM.

*KRAS* mutation in brain endothelial cells was sufficient to drive bAVMs in the postnatal stage. HA-tag and RAS^G12D^-expressing endothelial cells apparently formed dilated or tangled vasculatures, consistent with RAS^G12D^ expression in human cases (12). Human bAVMs have a tangled anastomosis of vessels containing arteriovenous shunting with multiple arteries converging and enlarged venous drainers (1, 4). The sources of afferent and efferent vessels are diverse, and fistula and aneurysms are frequently observed as an independent vascular architecture (4, 26). The present model well reproduced those structures. The multilayered vessels resembled those accumulating around the dilated vessel in human (30). The direct arteriovenous connections with multiple arteries and dilated veins, as well as aneurysms and enlarged vessels, corresponded well to the definition of a nidus. The model developed various sizes of lesions, estimated to range from 1,400 to 785,000 μm^2^, which is consistent with the variable size in human (The Virginia Radiosurgery AVM Scale or Spetzler-Martin grade; ref. 54). The sizes of the largest lesions were larger than those formed in young mice (17), again suggesting higher susceptibility in the postnatal brain. The growing state of endothelial cells or surrounding tissue environment in the postnatal brain might facilitate such a lesion generation, although the amount of induced KRAS may also affect this process.

### Pathological and molecular mechanisms.

The cellular and molecular mechanisms responsible for the vascular expansion and aberrant arteriovenous connections are not fully understood. There remains a debate about whether the malformation results from abnormal sprouting*,* cell size increase, proliferation, disorganized cell shape, arteriovenous connection, or/and progressive capillary dilation (39). Our model represented dilated, disorganized, and expanded endothelial cells, while the numbers of Ki67^+^ endothelial cells and the vascular density were limitedly changed. These results suggest that cellular growth, dilation, and abnormal connections rather than cell proliferation contribute to the malformation. This is consistent with the reports in cultured cells and the zebrafish model showing the increased size and expanded vessel diameter rather than proliferation (6, 16, 55). HA, RAS^G12D^, and pERK expressed in expanding endothelial cells support this notion. Abundant DEGs further suggest that capillaries rather than arteries and veins are involved in disease initiation. scRNA-Seq did not detect clear proliferative markers except *Ccnd1*, although proliferative angiogenesis would also be involved in part, as reported in human (18, 56–58) and mouse bAVM (17). Indeed, paradoxical to the increased lesion size, the number of capillaries was decreased, while undetermined cell types that lost intact mRNAs were increased. A part of malformed endothelial cells might degenerate due to abnormal intracellular activation or pathological environments such as hemorrhage and inflammation. scRNA-Seq further shows an increase of tip cells that usually exist until P8 in the cerebral cortex (59, 60). These cells might initiate abnormal vascular sprouting. Intriguingly, intravascular pillars were found in the enlarged lumens. An abortive intussusceptive angiogenesis might occur, which was proposed to obstruct blood flow and dilate vessels in cavernous vascular malformations (31). In any mechanism, an increase in blood flow pressure will further accelerate the expansion of the lumen and the lesion.

Although pERK expression was elevated as in human models and other models (6, 17, 21), exact molecular mechanisms that triggered cellular changes in growth, shape, or proliferation remain unknown. Previous studies in HUVECs showed that KRAS upregulated genes associated with angiogenesis, cell cycle, MAPK, and RNA processing, including Notch, VEGF, TGF-β–SMAD, and EndMT-related genes (6, 10, 15, 16, 61). Some pathways were also suggested in a mouse model (17), implying that KRAS stimulates or dysregulates angiogenic signaling and remodels endothelial cells. However, the present scRNA-Seq could not detect genes common in Notch and VEGF signaling (6, 15, 16) and EndMT-related genes (10, 15, 61). This would be consistent with no clear changes in human bAVMs (32, 39, 62). Rather, *Vegfa* expression was found to decrease in astrocytes (Supplemental Figure 5E). We only observed changes in TGF-β–related genes in capillaries, such as increase in *Spsb1*, a negative regulator of TGF-β (63), and a decrease in *Ltbp4*, which binds to TGF-β to control activation in the extracellular matrix. Decreased TGF-β signals were also observed in microglia/macrophage (Supplemental Figure 5D). Dysregulated TGF-β signals may be involved in the pathogenesis, as it is reported that HRAS inhibited TGF-β signals in endothelial cells (37). Different responses might be induced among the models such as in vitro versus in vivo and the endothelial cell types toward KRAS activation (64).

KRAS regulates multiple cellular processes, including vesicle trafficking, nuclear transport, cytoskeletal rearrangement, and cell cycle progression. Our scRNA-Seq data show changes in intracellular signaling and cytoskeletal molecules, suggesting that intrinsic signals, rather than extracellular growth factors, may drive the malformation. For example, *Rapgef5*, a guanine nucleotide exchange factor (GEF) that activates Rap GTPases, was increased while *Rap1a* was decreased both in our model and human bAVM (32). The Rap signals might be changed by KRAS activation (65, 66). Since Rap1 signal is important for VE-cadherin–mediated (CDH5-mediated) endothelial barrier junctions (67, 68), VEGFR2-mediated angiogenesis (69), and tubulogenesis (70), the dysregulation may cause changes in cytoskeleton, adhesion, or cell growth. Interestingly, Rap1a and Rapgef5 are proposed to be required for the nuclear import of β-catenin (*Ctnnb1*) for transcription (71). *Ctnnb1* is essential for BBB formation in Wnt signaling and was downregulated in our model and human bAVMs (32). This may lead to decrease in downstream molecules such as *Zic3* and *Foxq1* and in BBB transporters (*Slc2a1*, *Abcg2*, etc.) (72, 73), and it may correlate with disruption of tight junctions and BBB (74–76). This would lead to microhemorrhages in the present and other models (16, 17) and even in unruptured bAVMs in human (39).

How cellular and molecular interactions engage in bAVM development remains poorly understood (39). We applied comprehensive analyses with scRNA-Seq in a sporadic *KRAS* mutant model, in which a number of gene expression and cellular changes were observed. For example, abundant macrophage/microglia and astrocytes were reactivated, which would be caused by vascular leakage or hemorrhage. These cells may engage to repair and preserve the tissue, but they may also facilitate the malformation process. A recent study shows that resident macrophages enhance angiogenesis and monocyte infiltration in ALK1-deleted bAVM model in mice (77). In this context, upregulated *Ccl2* and *Ccl12*-Ccr2 pathways or/and *Mif* may mediate this process. Leukocytes were also increased as reported in mouse (77) and human bAVMs (2, 32, 62, 78), which may promote inflammation and lesion formation.

Although perivascular cells are reported to decrease in aneurysms or bAVMs (39, 76), we did not see a clear reduction in scRNA-Seq. We also could not find perivascular cells called AVM-specific mesenchymal cells that expressed CLDN5, MYL9, PDGFRB, and RGS5 in humans (10). On the other hand, a newly generated cluster was identified in the KRAS group, having distinct markers associated with the extracellular matrix and cell cycle, which may correspond to Ki67^+^ cells around the vasculature (Figure 4E). Since fibroblast-like cells are not present around capillaries (33), they might respond to disordered arteriovenous continuum and tissue repair signals and contribute to lesion formation.

Taken together, the analyses suggest multiple steps of malformation driven by *KRAS* mutation. KRAS induced exuberant growth in capillaries, which might lead to cytoskeletal disorganization of cytoskeleton and adhesion in endothelial cells and anastomosed arteriovenous connections. This would elevate blood flow, dilate vessels and form aneurysms, and make the barrier fragile, resulting in leakage and microhemorrhage. Inflammatory responses by microglia/macrophage and leukocytes, and subsequent astrocyte and fibrotic responses, were further induced. They would all be involved in the pathogenesis of lesion formation. Importantly, although the models in vitro are useful for understanding the mechanism of endothelial malformation and the efficacy of drugs (55, 79), they cannot reproduce the whole pathological processes above. The present model will offer an ideal preclinical model in this context. In addition, the present scRNA-Seq study provides a comprehensive database that is valuable to explore multiple cell type responses and overview the pathological process in disease development. The interactions of cells and molecules at different time points should be further examined to understand the pathogenesis and yield appropriate therapeutic targets.

### Therapeutic approach and future perspective.

Surgical approaches are difficult for large lesions and in eloquent or deep brain areas, which increase the risk of functional loss and mortality (1, 39). In unruptured bAVM, medical management rather than surgery may be superior for the prevention of death and stroke (80). Thus, multimodal treatments will help to suppress or delay the disease progress, ideally in the presymptomatic phase. They might also aid in suppressing recurrence or hemorrhage after the surgeries. The present mouse model showed the usefulness to test the efficacy of therapeutic methods.

MEK inhibitors SL327, U0126, or trametinib could reduce hemorrhage, shunt, and bAVM formation and improve survival rate in zebrafish and mouse models (6, 16, 17, 21). However, the effect is still partial, and in addition, systemic treatment might be compromised by causing side effects (81). The CRISPR/CasRx strategy employed here not only shows a superior therapeutic effect but also can reduce the risk of side effects, because of the specific target to the mutant sequence, as well as the restricted distribution. In this context, recently developed mutant KRAS inhibitors (13, 82) or genome editing of *KRAS* mutation may also be promising. Since we could not restrict the delivery to the malformed endothelial cells, additional improvements are required. AAV is useful for a long-standing effect compared with repeated treatments in drugs; indeed, 1 injection rescued the survival rate and suppressed malformation in our model. Combined treatment with MEK, KRAS, or BRAF inhibitors (11) could be further tested in the future.

The present study still has some limitations. For example, overexpression of mutant *KRAS* and virus infection may not entirely reflect the mechanisms of malformation in human bAVMs. Some leaked vascular lesions were further observed in the spleen, which should be improved by a more specific serotype of AAV and mouse lines. Second, since repeated AAV injections produce antibodies against AAVs to suppress infections (83, 84), the effect of subsequent therapeutic injections of AAV-CRISPR/CasRx might be underestimated. Even though technical and ethical problems must be overcome for gene editing and virus delivery in patients and lesion detection in the asymptomatic phase is also essential, the present study shows a proof of concept regarding the effect of CRISPR/CasRx viral delivery, which will contribute to develop novel therapeutic approaches.

In conclusion, the postnatal bAVM model established in this study highlights the pathological processes involved in neonatal bAVM development and demonstrates the potential of a novel therapeutic approach. The present flexible mouse model will serve as a valuable platform to further reveal pathological and molecular processes and to find optional therapeutic strategies for bAVMs.

## Methods

### Sex as a biological variable.

Male and female mice were used in the experiments, as we did not observe sex differences in generating vascular lesions (Supplemental Figure 2, A and B).

### Animals.

C57BL/6J mice (Charles River Laboratories), *Cdh5-CreERT2* (22), *lsl-Kras^G12D^* (008179, The Jackson Laboratory), *CAG*-*lox*-*CAT*-*lox*-*EGFP* (*lcl*-*EGFP*; from J. Robbins, Cincinnati Children’s Hospital Medical Center [CCHMC], Cincinnati) (85), and *CAG*-*lox*-*stop*-*lox*-*tdTomato* mice (*lsl*-*tdTomato*; Ai14; 007914, The Jackson Laboratory) were used in this study. The animals were randomly assigned to experimental groups.

### AAV production.

AAV plasmids were generated as follows. For pAAV-CAG-DIO-HA-KRAS^G12D^, HA-KRAS^G12V^ amplified from pLenti-PGK-KRAS4B(G12V) (35633, Addgene) by the primers (forward, 5′-CGGGATCCGCCACCATGTACCCATACGATGTTC-3′; reverse, 5′-CCCAAGCTTGCTGGGTCTTACATAATTACAC-3′) was subcloned into pcDNA3.1(-), and site-directed mutagenesis, KRAS c.35T>A, was performed with the primers (forward, 5′-GTGGTAGTTGGAGCTGATGGCGTAGGCAAGAGT-3′; reverse, 5′-ACTCTTGCCTACGCCATCAGCTCCAACTACCAC-3′). HA-KRAS^G12D^ was then amplified with primers (forward, 5′-CGGGATCCGCCACCATGTACCCATACGATG TTC-3′; reverse, 5′-CCCAAGCTTGCTGGGTCTTACATAATTACAC-3′) and subcloned into pAAV-CAG-DIO-MCS (multicloning sites) derived from pAAV-CAG-tdTomato (Penn Vector Core, AV-1-PV3365) in a substitution of tdTomato. For pAAV-CAG-HA-KRAS^G12D^ and pAAV-CAG-CasRx-Myc, HA-KRAS^G12D^ and CasRx-Myc amplified from pXR001:EF1a-CasRx-2A-EGFP (109049, Addgene) were subcloned into pAAV-CAG-tdTomato in a substitution of tdTomato. For pAAV-U6-gRNA-KRAS^G12D^, gRNA-KRAS^G12D^ (5′-TAGTTGGAGCTGATGGCGTAGG-3′) was subcloned into pXR003:CasRx gRNA cloning backbone (109053, Addgene), and U6-gRNA-KRAS^G12D^ was subcloned into HindIII-NotI sites of pAAV-CMV (6230, Takara). pAAV-CAG-EGFP (AV-1-PV1963, Penn Vector Core) and pAAV-Ple261-iCre (49113, Addgene) were further used.

To produce AAVs, human 293T cells (632273, TAKARA) were transfected with pAAV plasmid, pUCmini-iCAP-PHP.V1 (127847, Addgene) (24) or pXX2-187-NRGTEWD (pAAV-BR1) (23), and pHelper plasmid (TAKARA) (86). The supernatants were purified and concentrated in 0.001% Pluronic F68 (Thermo Fisher Scientific)/PBS. The titer was determined with a real-time PCR thermal cycler (Dice, TAKARA). AAV-BR1-CAG-EGFP (4.8 × 10^11^ GC/mL), AAV-PHP.V1-CAG-DIO-HA-KRAS^G12D^ (4.5 × 10^12^ GC/mL), AAV-PHP.V1-CAG-DIO-MCS (3.9 × 10^12^ GC/mL), AAV-PHP.V1-Ple261-iCre (5.4 × 10^12^ GC/mL), AAV-PHP.V1-CAG-CasRx-Myc (3.2 × 10^12^ GC/mL), and AAV-PHP.V1-U6-gRNA-KRAS^G12D^ (2.4 × 10^12^ GC/mL) were stored at −80°C until use.

### AAV and tamoxifen injections.

AAVs were injected through retro-orbital venous sinus at P5 under hypothermal anesthesia on ice. Tamoxifen (100 μg; in corn oil) was injected s.c. into the back. Four different models were tested in this study. (a) *Cdh5*-*CreERT2* mice crossed with *lsl*-*Kras^G12D^* mice (*Cdh5*-*CreERT2*;*lsl*-*Kras^G12D^*) were injected with tamoxifen at P5. (b) AAV-PHP.V1-Ple261-iCre (6 × 10^10^ GC) was injected into *lsl*-*Kras^G12D^*;*lcl*-*EGFP* mice at P5. (c) AAV-PHP.V1-Ple261-iCre and AAV-PHP.V1-CAG-DIO-HA-KRAS^G12D^ (6 × 10^10^ GC each) were simultaneously injected into *lcl*-*EGFP* mice at P5. (d) AAV-PHP.V1-CAG-DIO-HA-KRAS^G12D^ (6 × 10^10^, 1 × 10^9^, 5 × 10^8^, 1 × 10^8^, 5 × 10^7^, 3 × 10^7^, or 1 × 10^7^ GC) or control AAV-PHP.V1-CAG-DIO-MCS was injected into *Cdh5*-*CreERT2*;*lsl*-*tdTomato* mice with tamoxifen injection at P5. For CRISPR/CasRx experiments, AAV-PHP.V1-CAG-CasRx-Myc and AAV-PHP.V1-U6-gRNA-KRAS^G12D^ (1 × 10^10^ or 3 × 10^9^ GC) were injected retro-orbitally into *Cdh5*-*CreERT2*;*lsl*-*tdTomato* mice with AAV-PHP.V1-CAG-DIO-HA-KRAS^G12D^ (1 × 10^9^ or 3 × 10^7^) and tamoxifen simultaneously at P5 or at P19.

### Latex perfusion.

Blue latex (HX-Injection Medium, Holden’s latex) was infused from the left ventricle under anesthesia (17, 27). The brain was then fixed with 4% paraformaldehyde (PFA) overnight, dehydrated with methanol series (50%, 75%, 95%, and 100% each for 24 hours), and cleared in benzyl alcohol/benzyl benzoate (1:1 ratio). Images were acquired under a stereomicroscope (Olympus, SZX7).

### Three-dimensional analyses with clearing tissue.

Tissue clearing was performed by CUBIC with minor modifications (28). Postfixed brains were washed 3 times in PBS. For delipidation and decoloring, tissues were incubated in CUBIC-L (10 wt% *N*-butyldiethanolamine, 10 wt% Triton X-100) for 6 days at 37°C and washed 3 times in PBS for 2 hours each. The brain of AAV-PHP.V1-Ple261-iCre–injected *lsl*-*Kras^G12D^*;*lcl*-*EGFP* mouse was incubated with FITC conjugated mouse anti–α-SMA antibody (1:100, F3777, Sigma-Aldrich) in the staining buffer (0.5% polyethylene glycol mono-p-isooctylphenyl ether, 0.25% casein, 0.01% sodium azide; Nacalai Tesque) for 5 days at room temperature. They were then washed 3 times in 0.05% NaN_3_/PBS for 1 hour each. For refractive index matching, they were incubated in 50% CUBIC-R (45 weight percentage [wt%] antipyrine, 30 wt% nicotinamide [pH 8–9], adjusted by N-butyldiethanolamine) for 6 hours and then in CUBIC-R for 24 hours. The cleared tissues were imaged under a light-sheet microscopy (MVX10-LS, Olympus or Blaze, Miltenyi Biotec). The obtained images were reconstructed in IMARIS software, and the vessels were traced by Filament Tracer tool.

### IHC.

The animals were perfused with 4% PFA in 0.1M phosphate buffer. The brain and spinal cord were postfixed in the same fixatives between 2 hours and overnight. The tissues were immersed in 30% sucrose/PBS overnight and embedded in Tissue-Tek OCT compound (Sakura Finetek). Serial 20 or 50 μm–thick sections were made with a cryostat and mounted on MAS-coated slides (Matsunami). H&E and iron staining (IRN-1, ScyTek) were performed by standard protocols. For IHC, the sections were blocked with 1% BSA in 0.3% Triton X-100/PBS for 2 hours and incubated with primary antibodies 1 or 2 overnight periods at 4°C. The following antibodies were used: goat anti-mCherry (1:500; AB0040, Sicgen), rabbit anti-RFP (1:1000; 600-401-379, Rockland), rabbit anti-GFP (1:1000; A11122, Invitrogen), rat anti-GFP (1:1000; 04404-84, Nacalai), rabbit anti-NeuN (1:500; ABN78, Merck), rabbit anti–phospho-ERK (1:250; 9101, Cell Signaling Technology), rat anti-HA (1:500; 11867423001, Roche), rabbit anti-Ki67 (1:500; MA5-14520, Thermo Fisher Scientific), rabbit anti-RasG12D (1:100; ab221163, Abcam), rabbit anti-Iba1 (1:500; 019-19741, Wako), mouse anti-GFAP (1:400; G3893, MilliporeSigma), goat anti-CD31 (1:50; AF3628, R&D Systems), rat anti-CD31 (1:50; DIA-310, Dianova), rat anti-CD3 (1:500; 555273, BD Biosciences), rat anti-CD45R (B220; 550286, BD Biosciences), rabbit anti-MPO (1:200; RB-373, Thermo Fisher Scientific), rat anti-Ly6G (1:200; 127601, BioLegend), rabbit anti-RAPGEF5 (1:250; ab129008, Abcam), and rabbit anti-SPSB1 (1:30; PA5-89504, Thermo Fisher Scientific) antibodies. After washing with 0.1% Triton X-100/PBS, the sections were incubated with secondary antibodies for 2 hours: Alexa Fluor 488, 568, or 647 donkey anti-rabbit, -mouse, -rat, and -goat IgG antibodies (1:1,000; Invitrogen). The sections were counterstained with DAPI (1 μg/mL; Santa Cruz Biotechnology Inc.) to visualize nuclei. The images were acquired with a fluorescence microscope (Olympus, BX51) or a confocal microscope (Olympus, FV3000).

For histological evaluation, the number of vascular lesions was counted in tdTomato-labeled brain sections, and the diameter of lesions and vessels were assessed by ImageJ software (NIH). The ratio of HA^+^ endothelial cells in the lesion was assessed in 3 brain sections per mouse. pERK and SPSB1 expression were evaluated by mean gray values in endothelial cells of 3 lesions and adjacent control areas per mouse. Ki67^+^ cells in tdTomato^+^ endothelial cells and the cells in the parenchyma of the lesion were counted in 11–26 serial sections per mouse. Cell and nuclear area of tdTomato^+^ endothelial cells and Iba1^+^ and GFAP^+^ cell areas were evaluated in 3 lesions and adjacent control areas per mouse. Ly6G^+^ and B220^+^ cell density were assessed in 4–12 lesions and adjacent control areas per mouse. RAPGEF5 expression was evaluated by mean gray values in the nuclei of endothelial cells in 5–6 lesions and adjacent control areas.

### Cell isolation and scRNA-Seq.

scRNA-Seq were performed for *Cdh5*-*CreERT2*;*lsl*-*tdTomato* mice injected with AAV-PHP.V1-CAG-DIO-HA-KRAS^G12D^ (1 × 10^9^ GC) or control AAV-PHP.V1-CAG-DIO-MCS (1 × 10^9^ GC) and tamoxifen at P5. Cerebral cortices were dissected out at P13 and P20, chopped into small pieces with scissors, and incubated for 45 minutes at 22 °C in a 5% pronase solution containing 0.5 mM GlutaMAX and 15 μM Actinomycin D in Hibernate A. The specimens were further dissociated with a Pasteur pipette and filtered over a 70 μm mesh to remove undigested tissues. Cells were collected by 300*g* centrifugation for 10 minutes at 4°C. The pellet was resuspended in PBS, and cell debris was removed by Debris Removal Solution (Miltenyi Biotec). The final cell pellet was resuspended in Hibernate A and tdTomato^+^ endothelial cells, and tdTomato^–^ cells were sorted by FACS (BD Biosciences FACSAria III). Dead cells were labeled by LIVE/DEAD Fixable Violet Dead Cell Stain Kit, for 405 nm excitation (Invitrogen). Approximately 20,000 tdTomato^+^ viable and 80,000 tdTomato^–^ viable cells were collected, mixed, and kept at –80°C until library preparation. The scRNA-Seq libraries were prepared using Chromium Single Cell 3′ Reagent Kits v3.1 and Chromium Controller (10X Genomics). A 28/96 bp pair end sequencing of the libraries was performed by NovaSeq 6000 (Illumina).

### scRNA-Seq data processing.

The sequenced data were mapped to the prebuilt mouse genome reference (mm10) using Cell Ranger software v6.0.0 and v6.1.2 (10X Genomics) after adding custom sequences of *tdTomato*. We first removed ambient mRNAs by SoupX (87) and doublet cells by DoubletFinder (88), and then we removed cells that had mitochondrial genes at greater than 10% occurrence among detected genes, total UMI count less than 500, UMI count above or below the 3 median absolute deviations from the median value, and total genes fewer than 200. Normalization, dimensionality reduction, cell clustering, and visualization were performed by Seurat (v.4) (89). DEGs were determined by FindMarkers in Seurat package with a cut-off value of adjusted *P* < 0.05. Sex chromosome genes showing sex-biased expressions (e.g., *Uty*, *Ddx3y*, *Eif2s3y*, *Xist*, *Tsix*) were excluded from the interpretation. Pathway and GO analysis were performed by SCPA (v.1.5.3) and clusterProfiler (v.4) with default parameters (90, 91). We utilized seurat_extract function of SCPA to extract gene expressions of endothelial cells from the control and KRAS groups. Pathway analysis was performed by the compare_pathways function and the hallmark gene set collections.

### CRISPR-CasRx in cell culture and quantitative PCR (qPCR).

gRNAs with the spacer covering the region of mutated nucleoside A of *KRAS^G12D^*, 5′-TAGTTGGAGCTGATGGCGTAGG-3′ was selected as previously reported (47). HEK293T cells were transfected with pAAV-CAG-HA-KRAS^G12D^, pAAV-CAG-CasRx-Myc, and pAAV-U6-gRNA-KRAS^G12D^ at 1:300:300 with Lipofectamine 3000 (Thermo Fisher Scientific) and cultured in 10% FBS/DMEM at 5% CO_2_, 37°C for 2 days. Cell lysis and mRNA collection were performed with RNeasy kit (QIAGEN). The total RNA underwent reverse transcription to make cDNAs by PrimeScript RT reagent Kit (TAKARA). Reaction mixture of 25 μL contained 12.5 μL TB Green Fast qPCR Mix (TAKARA), 1 μL each of the sense and antisense primers (10 μM), and 2 μL cDNA was treated with 40 amplification cycles (denaturation at 95°C for 5 seconds, annealing and extension at 60°C for 10 seconds) in real-time PCR thermal cycler (Dice, TAKARA). Real-time PCR was performed with primer sets of human *KRAS* forward, (5′-GGCAAGAGTGCCTTGACGATA-3′) and reverse (5′-TTGACCTGCTGTGTCGAGAAT-3′); and β-*actin* (*ACTB*) forward (5′-CACCATTGGCAATGAGCGGTTC-3′) and reverse (5′-AGGTCTTTGCGGATGTCCACGT-3′). Relative *KRAS* mRNA expression normalized with *ACTB* was calculated with the Ct and ΔΔCt method.

### Trametinib treatment.

Trametinib (HY-10999, MedChemExpress; 1 mg/kg) in 0.5% hydroxypropyl methylcellulose and 0.2% Tween-80 was administered daily by oral gavage (17, 21) for 2 weeks from the day of AAV-PHP.V1-CAG-DIO-HA-KRAS^G12D^ injection.

### Statistics.

Quantitative data are represented as the mean ± SEM if they are not indicated in the figure legends, or as the mean ± SD when they are noted in the figure legends. Statistical analyses were performed with Prism 7 (GraphPad). Differences among groups were analyzed by 1-way ANOVA followed by Tukey’s test, Kruskal-Wallis test followed by Dunn’s multiple-comparison test, or 2-way repeated-measures ANOVA followed by Bonferroni’s test. Differences of 2 groups were analyzed by 2-tailed unpaired *t* test or Mann-Whitney *U* test. Survival curves were compared with the log-rank test. A *P* value less than 0.05 was considered statistically significant.

### Study approval.

All the procedures for mouse experiments were performed in accordance with protocols approved by the IACUC of Niigata University.

### Data availability.

Data used in the figures and supplemental figures are available in the Supporting Data Values file. All raw sequence files of scRNA-Seq are available from DDBJ (accession nos. DRA015668 and DRA013415).

## Author contributions

SS, MN, and MU conceived the project and designed the experiments. SS, YN, and MU performed the experiments and analyzed the data. SM performed scRNA-Seq analyses. TS, KH, and M Okada supported experiments. HH, M Oishi, and YF supervised the studies. JK supported AAV experiments. YK supported mouse line experiments. KT supported clearing tissue experiments. SS, MN, and MU wrote the manuscript. SS and YN share first authorship due to their equal contribution to the experiments. SS is listed first because of the leading role in the study.

## Supplementary Material

Supplemental data

Supplemental table 1

Supplemental table 2

Supplemental table 3

Supplemental table 4

Supplemental table 5

Supplemental table 6

Supplemental table 7

Supplemental table 8

Supplemental table 9

Supplemental table 10

Supplemental video 1

Supplemental video 2

Supporting data values

## Version 1 11/22/2024

Electronic publication
