# Supplementary material for: CRISPR/CasRx suppresses KRAS-induced brain arteriovenous malformation developed in postnatal brain endothelial cells in mice
Source: JCI Insight. 2024 Nov 22;9(22):e179729. doi: 10.1172/jci.insight.179729 (PMC11601911; doi:10.1172/jci.insight.179729)
Supplement: Supplemental data [file jciinsight-9-179729-s238.pdf]

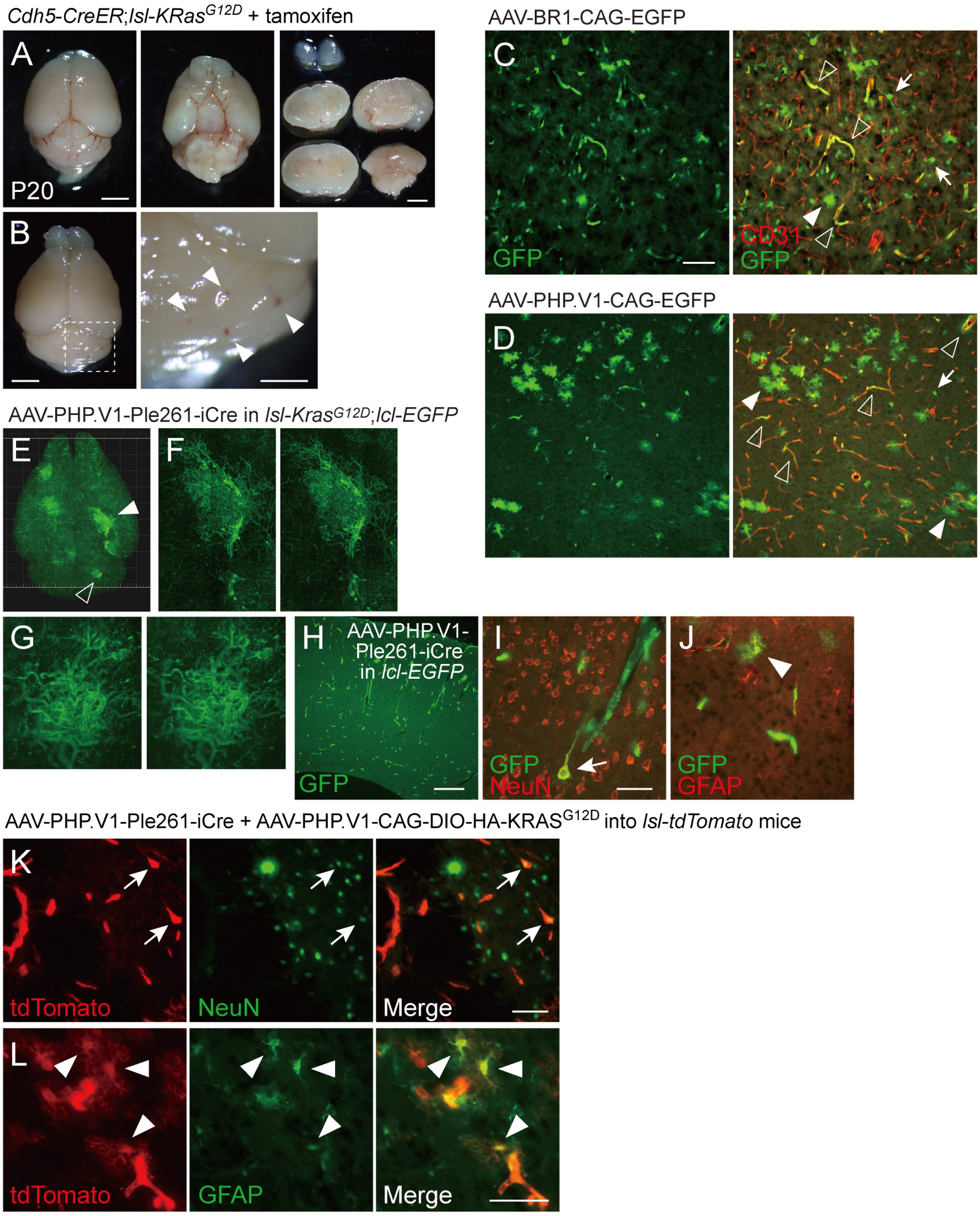

Supplemental Figure 1. Evaluation of mouse bAVM models in the postnatal stage. (**A**, **B**) Brain images of two representative *Cdh5-CreERT2*;*lsl-Kras^{G12D}* mice (P20) injected with tamoxifen at P5. One mouse showed small hemorrhagic spots at the cerebellum (**B**; the right panel is the magnified view of the dotted area). (**C**, **D**) Representative brain section images of P21 mice, which were retro-orbitally injected with AAV-BR1-CAG-EGFP (**C**) or AAV-PHP.V1-CAG-EGFP (**D**) at P5. GFP, green; endothelial cell marker CD31, red. Open arrowheads, endothelial cells;

white arrowheads, astrocytes; arrows, neurons. (**E**–**G**) 3D images of a cleared brain of AAV-PHP.V1-Ple261-iCre-injected *lsl-Kras*^{G12D};*lcl-EGFP* mouse, showing vascular tangle formation (arrowheads) visualized with GFP and α-SMA staining (green). (**F**) and (**G**) are magnified views of an closed and open arrowhead of (**E**), respectively. (**H**–**J**) Representative brain section image of AAV-PHP.V1-Ple261-iCre-induced *lcl-EGFP* mice (6 x 10^{10} GC), showing ectopic EGFP expressions in NeuN$^+$ neurons (**I**, arrow) and GFAP$^+$ astrocytes (**J**, arrowhead). (**K**, **L**) Ectopic tdTomato expressions in NeuN$^+$ neurons (**K**, arrows) and GFAP$^+$ astrocytes (**L**, arrowheads) in a model of AAV-PHP.V1-Ple261-iCre and AAV-PHP.V1-CAG-DIO-HA-KRAS^{G12D} injection in *lsl-tdTomato* mice (single color images of Figure 1M, N). Scale bars; 2 mm (**A**, the left panel of **B**), 1 mm (the right panel of **B**), 100 μm (**C**, **D**), 250 μm (**H**), 50 μm (**I**–**L**).

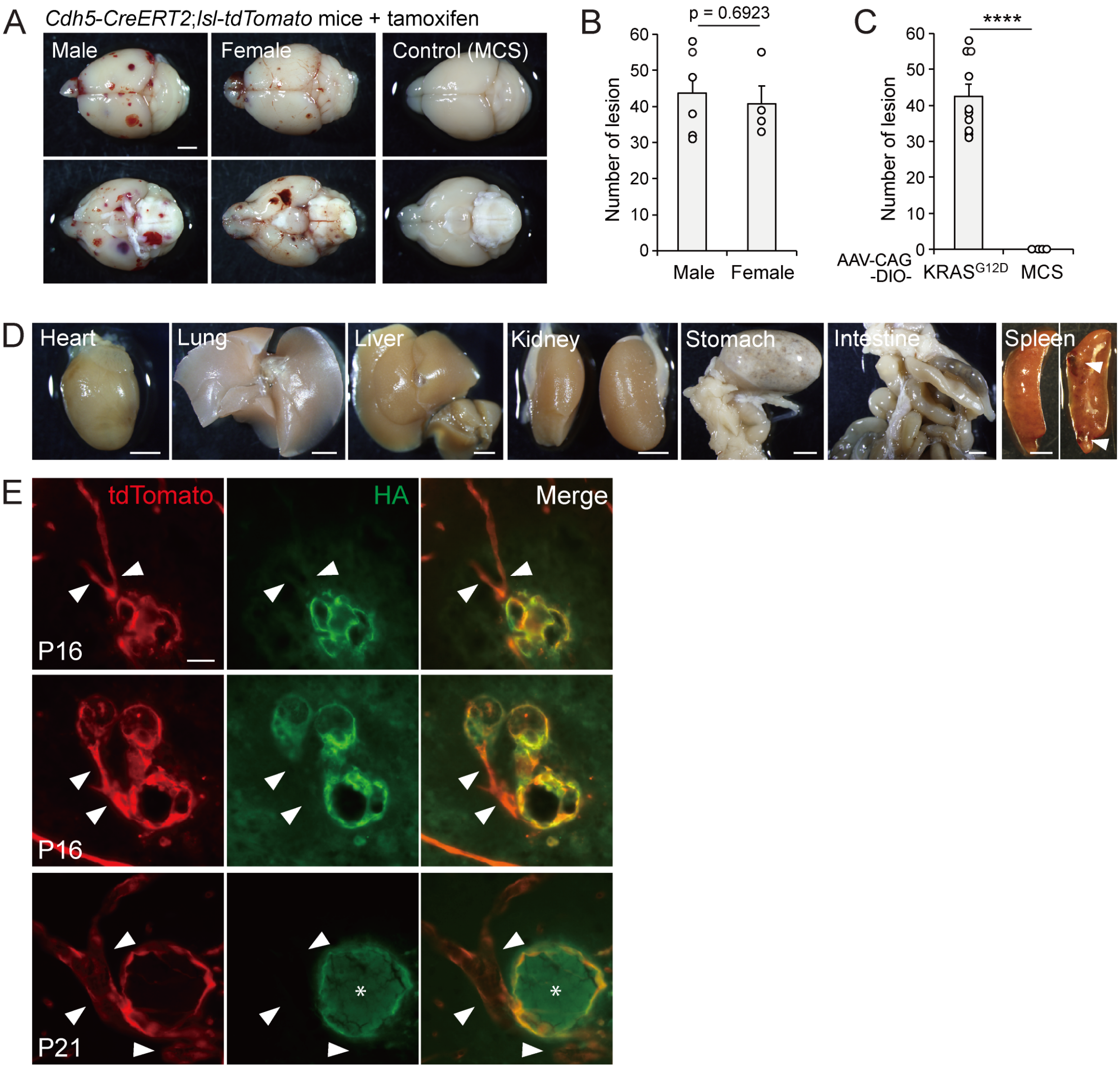

Supplemental Figure 2. Evaluation of vascular lesions in KRAS^{G12D}-induced *Cdh5-CreERT;lsl-tdTomato* mice. (**A**) Representative brain images of male and female of *Cdh5-CreERT;lsl-tdTomato* mice injected with AAV-CAG-DIO-KRAS^{G12D} or control AAV-CAG-DIO-MCS (1×10^9 GC, P21). (**B**) The number of lesions in males (n = 6) and females (n = 4). Unpaired t-test. (**C**) The number of lesions in AAV-CAG-DIO-KRAS^{G12D} (n = 10) and control AAV-CAG-DIO-MCS (n = 4)-injected *Cdh5-CreERT;lsl-tdTomato* mice. ****$P < 0.0001$, unpaired t-test. (**D**) Representative images of organs of *Cdh5-CreERT;lsl-tdTomato* mice injected with AAV-CAG-DIO-KRAS^{G12D} (1×10^9 GC, P21). Arrowheads, hemorrhagic sites in the spleen. (**E**) Representative images of HA-negative tdTomato$^+$ endothelial cells located in the edge of the lesion in *Cdh5-CreERT;lsl-tdTomato* mice injected with AAV-CAG-DIO-KRAS^{G12D} (arrowheads, 1×10^9 GC, P16 and P21). The upper images are high magnifications of Figure 2L. Asterisk, nonspecific signals of thrombus. Scale bars; 2 mm (**A**, **D**), 25 µm (**E**).

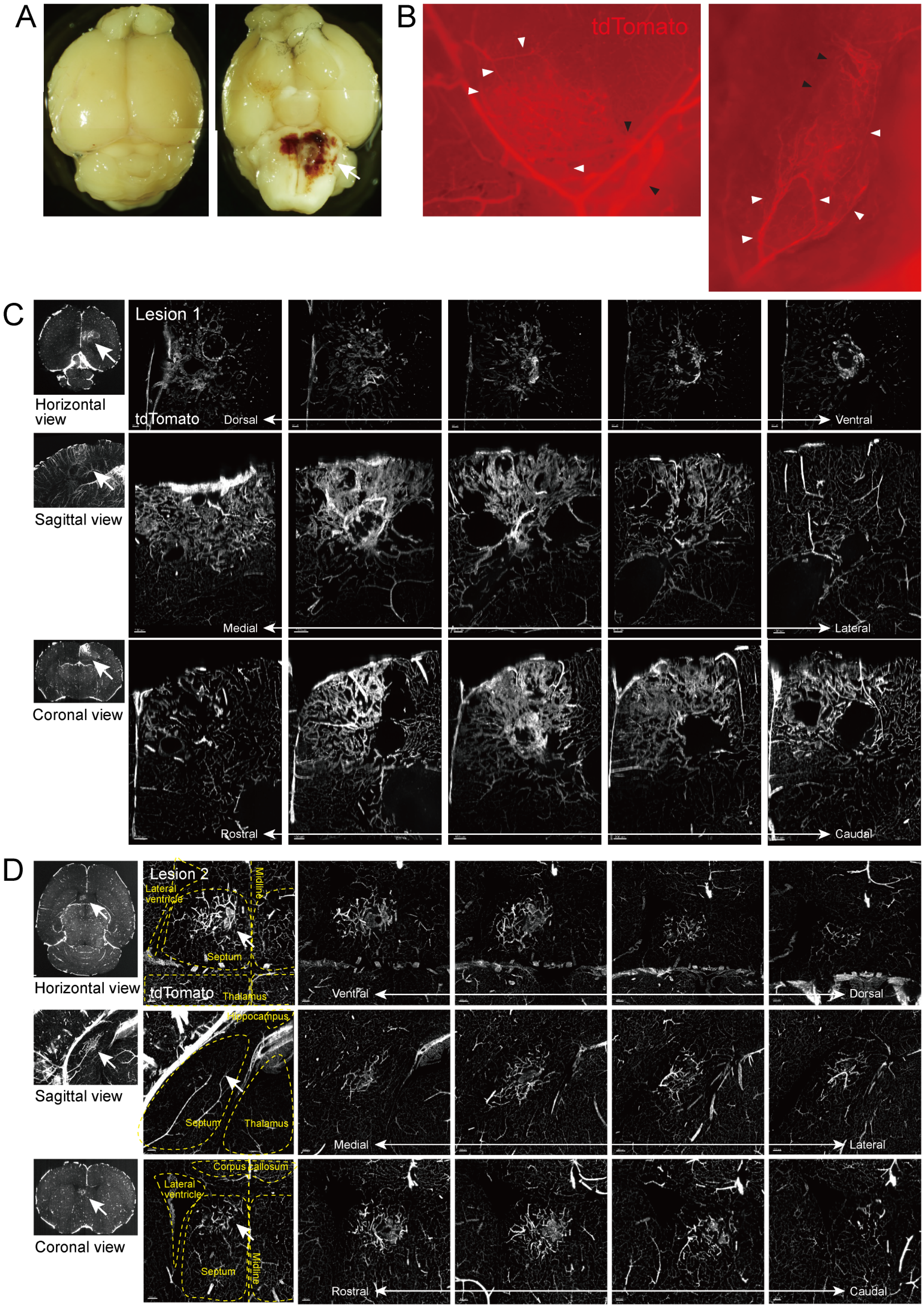

Supplemental Figure 3. Observations of vascular tangles formed in KRAS^{G12D}-induced *Cdh5-CreERT;lsl-tdTomato* mice. (**A**) Representative images of hemorrhagic lesion formed in

the brain stem of AAV-CAG-DIO-KRAS^{G12D}-injected *Cdh5-CreERT*;*lsl-tdTomato* mice (3×10^7 GC, P42, an arrow). Two images were combined in each panel. (**B**) Fluorescent observation of the hemorrhagic area of (**A**, an arrow) in stereomicroscopy (white arrowheads, feeding arteries; black arrowheads, dilated veins). (**C**) Serial section images of lesion 1 (arrows) of the clearing brain in horizontal (upper panels), sagittal (middle panels), and coronal planes (bottom panels). (**D**) Serial section images of lesion 2 (arrows) of the clearing brain in horizontal (upper panels), sagittal (middle panels), and coronal planes (bottom panels).

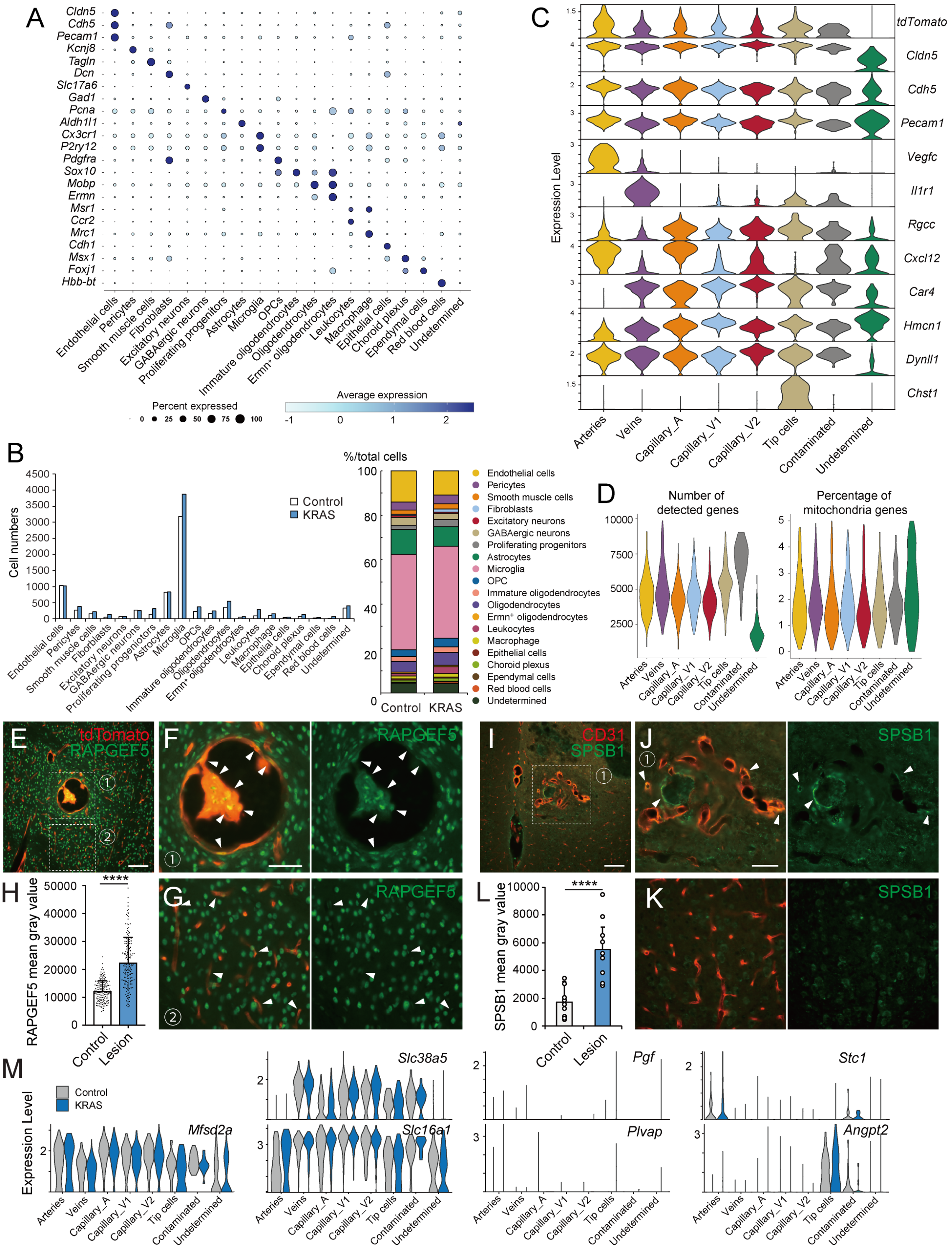

Supplemental Figure 4. Single-cell RNA-seq data of total brain and endothelial cells in KRAS^{G12D}-induced mice. (**A**) Marker gene expressions in each cluster of brain cells (related to Figure 5A). (**B**) Cell numbers (right graph) and ratio (left graph) of the brain cell types in control and KRAS-group. (**C**) Marker gene expressions in endothelial cell types. (**D**) Lower numbers of detected genes and increased percentage of mitochondria-related genes in “undetermined” cluster

of endothelial cells. (**E**–**G**) RAPGEF5 expression (green) in tdTomato$^+$ endothelial cells (red) in the lesion (**F**) and adjacent intact area (**G**) (magnified views of the dotted areas in **E**). Arrowheads, RAPGEF$^+$ nuclei. (**H**) RAPGEF5 expression levels in tdTomato$^+$ endothelial cells in the control area and lesions. Mean ± SD, ****P < 0.0001 (n = 157, 165 nuclei in three animals), Mann-Whitney test. (**I**–**K**) SPSB1 expression (green) in CD31$^+$ endothelial cells (red) in the lesion (**J**, a magnified view of the dotted area in **I**) and adjacent intact area (**K**). Arrowheads, SPSB1$^+$ endothelial cells. (**L**) SPSB1 expression levels in CD31$^+$ endothelial cells in the control area and lesions. Mean ± SEM, ****P < 0.0001, n = 9 lesions in three animals, unpaired t-test. (**M**) Expressions of genes reported to be up- or downregulated in human nidus cluster (32). Scale bars; 100 μm (**E**, **I**), 50 μm (**F**, **G**, **J**, **K**).

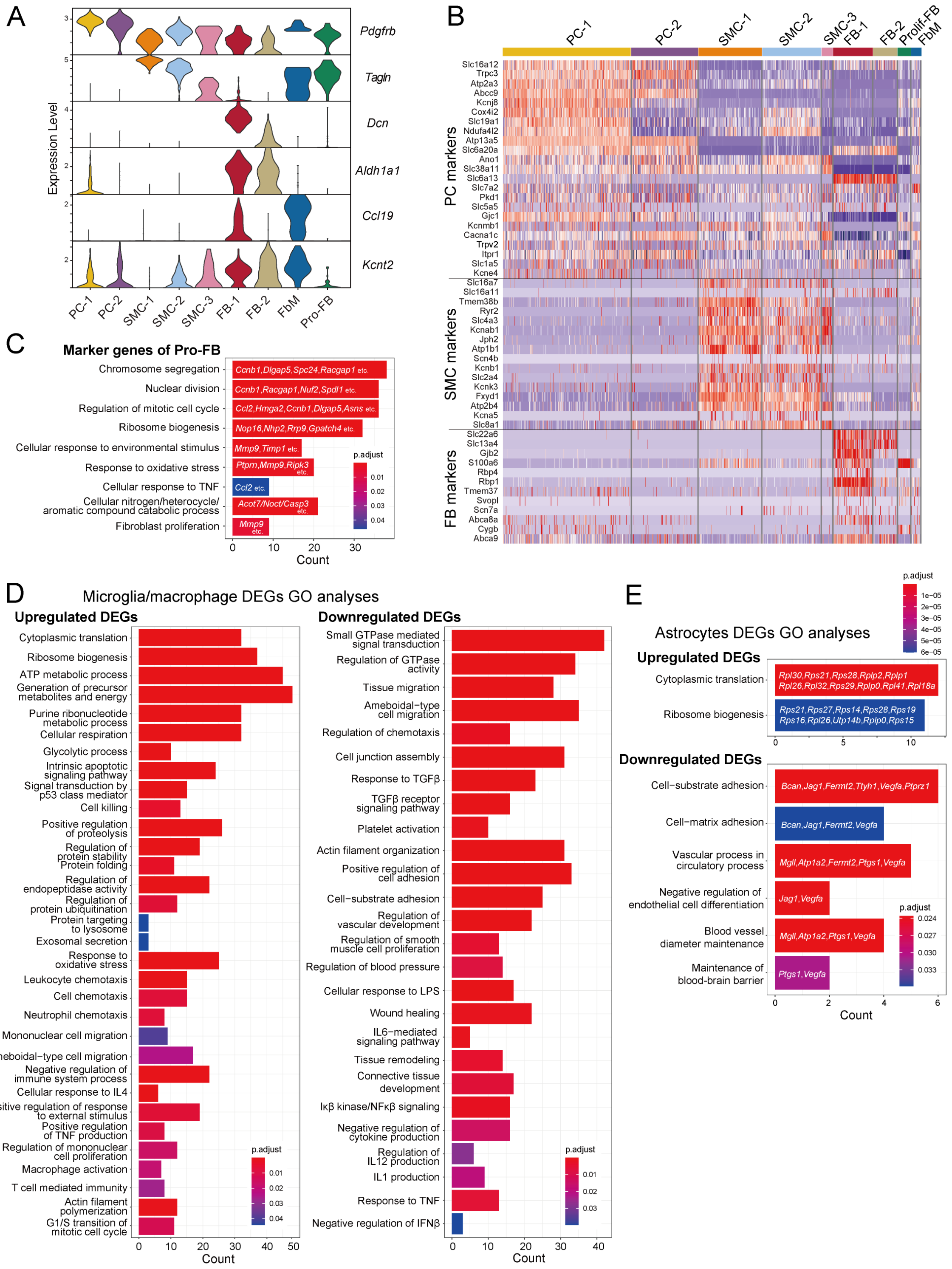

Supplemental Figure 5. Single-cell RNA-seq data of perivascular and glial cells in KRAS^{G12D}-induced mice. (**A**) Marker gene expressions in perivascular cell types. *Pdgfrb*, pericyte marker; *Tagln*, smooth muscle cell marker; *Dcn* and *Aldh1a1*, fibroblast-like cell markers; *Ccl19* and *Kcnt2*, fibromyocyte markers (32). (**B**) Expressions of marker genes for pericytes, smooth muscle cells and fibroblast-like cells (33) in each cluster of perivascular cells

(related to Figure 6A). (**C**) Representative GO terms of marker genes for proliferative fibroblast-like cells. (**D**) Representative GO terms in up- and downregulated DEGs in microglia/macrophage (KRAS vs control). (**E**) Representative GO terms in up- and downregulated DEGs in astrocytes (KRAS vs control).

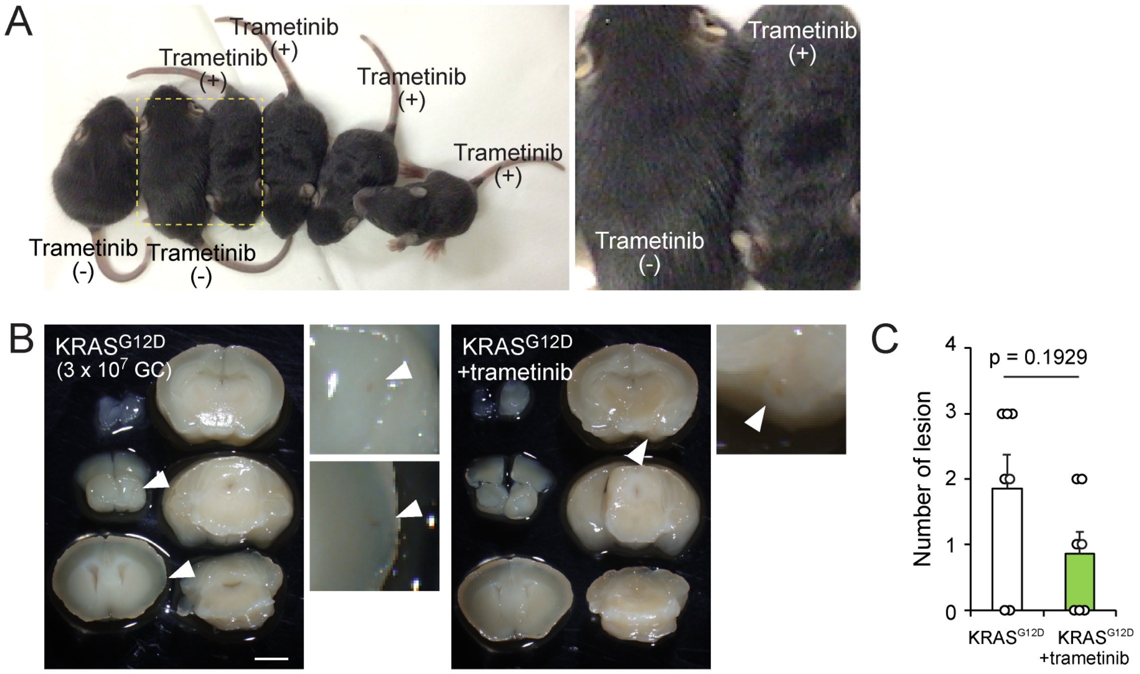

Supplemental Figure 6. Trametinib treatment in KRAS^{G12D}-induced mice. (**A**) Representative images of the mice treated with a MEK inhibitor trametinib (n = 4) and non-treated controls (n = 2) for 2 weeks (P19). Right panel is a higher magnification of the dotted area in the left one. Note a smaller body and wooly hair in the trametinib-treated mice. (**B**) Representative brain images of *KRAS^{G12D}*-induced (3 × 10^7 GC) and trametinib-treated mice (1 mg/kg, daily for 2 weeks) at P19. Right panels are magnified views of the lesion (arrowheads). (**C**) The number of lesions in KRAS^{G12D}-induced (3 × 10^7 GC) and trametinib-treated mice. n = 7, Mann-Whitney test. Scale bar, 2 mm.

Supplemental Table 1. Marker genes of endothelial cell types.

Supplemental Table 2. DEGs in endothelial cell types (KRAS vs control group).

Supplemental Table 3. GO analyses of DEGs in capillaries.

Supplemental Table 4. DEGs common in endothelial cells of mouse and human bAVMs.

Supplemental Table 5. Marker genes of perivascular cell types.

Supplemental Table 6. GO analyses of proliferative FB.

Supplemental Table 7. Marker genes of microglia/macrophage clusters.

Supplemental Table 8. DEGs in microglia/macrophage (KRAS vs control group).

Supplemental Table 9. Marker genes of astrocyte clusters.

Supplemental Table 10. DEGs in astrocyte clusters (KRAS vs control group).

Supplemental Movie 1. Three-dimensional structures of lesion 1 in cleared brains of $KRAS^{G12D}$-induced *Cdh5-CreERT*;*lsl-tdTomato* mice.

Supplemental Movie 2. Three-dimensional structures of lesion 2 in cleared brains of $KRAS^{G12D}$-induced *Cdh5-CreERT*;*lsl-tdTomato* mice.
